# Hydrogels from Renewable Resources: Advances in 3D Networks Based on Cellulose and Hemicellulose

**DOI:** 10.3390/polym17202760

**Published:** 2025-10-15

**Authors:** Diana Elena Ciolacu

**Affiliations:** Department of Natural Polymers, Bioactive and Biocompatible Materials, “Petru Poni” Institute of Macromolecular Chemistry, 700487 Iasi, Romania; dciolacu@icmpp.ro

**Keywords:** cellulose, hemicellulose, hydrogels, crosslinking, structure, properties, biomedical applications

## Abstract

In recent years, natural polymers have gained significant attention due to their abundance, biodegradability and versatility, offering a promising alternative to conventional synthetic polymers. Among natural polymers, cellulose and hemicellulose hold a special place, being the most abundant plant polysaccharides in nature, which serve as key structural materials in the synthesis of hydrogels. Cellulose has attracted significant attention in the development of hydrogels due to the fact that it confers desirable mechanical properties, high water absorption and biocompatibility. Hemicellulose, although with a more amorphous structure than cellulose, contains various functional groups that facilitate its chemical modification. With an environmentally friendly nature and low cost, these polysaccharides have gained major interest and are highly appreciated by both the academic and industrial communities. This review comprehensively presents recent advances in the design and development of hydrogels made from renewable biopolymers—cellulose and hemicellulose—providing an in-depth exploration of the information recorded over the past five years. The latest strategies for the synthesis of hydrogels, their formation mechanisms and their most important properties are analyzed and summarized in detail from the perspective of physical and chemical crosslinking. A comparative analysis is performed between these hydrogels, highlighting not only the advantages and disadvantages of each type of hydrogel but also the main challenges associated with the balance between mechanical strength, swelling capacity, biodegradability and cost-effectiveness. Finally, the advanced biomedical applications of these hydrogels in areas such as drug delivery, wound dressings and tissue engineering are presented in detail.

## 1. Introduction

The increasing demand for lignocellulosic biomass resources has led to the evaluation of their utilization possibilities and the production of value-added materials for a wide range of applications, focusing on the development of cost-effective syntheses [1]. Lignocellulosic biomass is a renewable and abundant feedstock with enormous potential for a large number of applications for human sustainability, such as chemicals, biofuels, biomolecules and biomaterials [2].

Lignocellulosic biomass origin can be classified into the following categories: (i) woody biomass: softwood, hardwood and recycled wood fibers; and (ii) non-woody biomass: agriculture crops, industrial waste and urban waste [3]. Biopolymers with specific properties like abundant availability, renewability, lack of toxicity and biodegradability can be obtained from different renewable resources such as (i) polymers extracted from biomass: polysaccharides, proteins and lipids; (ii) polymers synthesized from bio-derived monomers: polylactides and other polyesters; and (iii) polymers produced directly by the natural or genetically modified organism: polyhydroxyalkanoates and bacterial cellulose [4]. Lignocellulosic biomass, an abundant and renewable resource from plants, is mainly composed of cellulose, hemicelluloses and lignin.

*Cellulose*, the most abundant polysaccharide on Earth, can be found in the cell walls of the wood or different fiber plants (cotton, jute, hemp, etc.), can be extracted from algae or can be produced by bacteria. Even though cellulose is abundant and cost-effective, it often requires extensive purification to remove lignin and hemicellulose. On the other hand, bacterial cellulose (BC), produced by microorganisms like *Gluconacetobacter xylinum*, has a higher purity, crystallinity and water holding capacity, making it particularly advantageous for biomedical applications [5,6]. *Hemicelluloses*, the second most abundant group of plant polysaccharides, have a branched, heterogeneous structure that is more amorphous than cellulose, which forms complex networks between cellulose and lignin, providing structural strength to plant cell walls [7,8]. In addition to their advantages, such as their wide availability and low cost, hemicelluloses also present major disadvantages that hinder their widespread application, such as limited extraction efficiency and poor mechanical properties [9].

Given the above-mentioned distinct characteristics of cellulose and hemicelluloses, it is obvious that they represent promising natural precursors for the preparation of hydrogels. Their attributes, together with the possibility of customizable functionalization, recommend them for obtaining hydrogels that present biocompatibility, biodegradability, a structure similar to the extracellular matrix (ECM) and remarkable mechanical properties, as well as the possibility of controlled drug release and the ability to promote cell growth [10]. *Cellulose-based hydrogels* have garnered significant attention due to their excellent water retention capacity, mechanical robustness and economic feasibility. These properties recommend them as particularly suitable for diverse applications. In the biomedical field, cellulose-based hydrogels have distinguished themselves as being suitable for uses such as controlled drug release and wound healing, and as scaffolds for tissue engineering (TE) [11]. *Hemicellulose-based hydrogels* stand out for their cost-effectiveness, but also for the possibility to design hydrogels with specific properties, such as antitumor efficacy [12,13], immune system modulation [14] and antioxidant properties [15], opening an important direction for the high-value use of hemicelluloses.

All this together recommends them for applications in various fields such as biomedicine (drug delivery, wound dressings, tissue engineering and biosensors) [16,17,18,19,20], environmental protection (absorption of heavy metal ions, dyes and pesticides) [21], agricultural (water retention and pesticide delivery) [22,23], art conservation [24] and flexible electronics [25,26].

**Figure 1:**
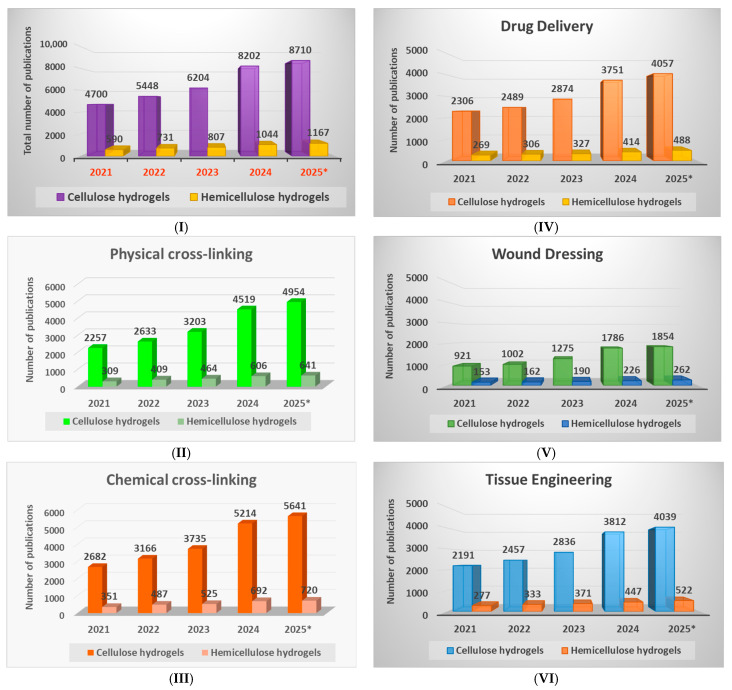
The number of publications from 2021 to 2025 using the following topic keywords: (**I**) “Cellulose hydrogels” and “Hemicellulose hydrogels”, as well as (**II**) “Physical cross-linking”; (**III**) “Chemical cross-linking”; (**IV**) “Drug Delivery”; (**V**) “Wound Dressing”; and (**VI**) “Tissue Engineering”. Searches were conducted in the Web of Science databases in October 2025 (2025*).

Taking into account the aspects mentioned above, a detailed investigation of the scientific interest in this topic was carried out and the number of publications for each type of hydrogel (cellulose- and hemicellulose-based hydrogels) published in the last five years was searched (Figure 1I). The searches were conducted in the main collection of the online database Web of Science (WOS) for the period 2021–2025 in October 2025. It should be noted that for the year 2025, the number of published articles does not represent the total number for this year, but rather only the number of articles published until October 2025, which is mentioned in the figures as 2025*.

A growing trend in the scientific interest in this field was observed through the increased number of publications from 2021 to 2025, with greater interest in cellulose-based hydrogels (33,264), followed by hemicellulose-based ones (4339).

Furthermore, in order to highlight the interest of researchers in a certain type of crosslinking method used in the hydrogels’ preparation, the keywords “physical cross-linking” (Figure 1II) and “chemical cross-linking” (Figure 1III) were searched for the same two types of hydrogels. A similar trend was observed also in this case, with a greater interest in cellulose-based hydrogels, with the mention that the number of publications related to the chemical crosslinking process (23,213) was slightly higher than those related to the physical crosslinking process (19,995).

Also, in the last five years, there has been a sustained increase in scientific interest in the biomedical applications of cellulose- and hemicellulose-based hydrogels, with a more sustained scientific activity compared to cellulose-based hydrogels, recording a number of articles 88% higher than hemicellulose-based hydrogels. To highlight the scientific interest in each medical field, the number of publications was searched for in the following key areas: “drug delivery” (Figure 1IV), “wound dressings” (Figure 1V) and “tissue engineering” (Figure 1VI). It was observed that scientific interest is oriented approximately equally towards the field of tissue engineering (17,285) and controlled drug delivery systems (17,281), followed by the field of wound dressings (7831).

Given the high interest of the scientific world in this subject, we considered it of interest to review the latest achievements from the last five years (2021–2025) regarding the synthesis of cellulose- and hemicellulose-based hydrogels and their characteristics, as well as an in-depth exploration of their use in the field of biomedical applications.

To our knowledge, there is no such comparative study that discusses the problems and potential solutions related to cellulose- and hemicellulose-based hydrogels and highlights the most recent discoveries (2021–2025) related to these hydrogels.

This review provides a comprehensive analysis of the latest advances in the field of hydrogels based on the most abundant plant polysaccharides in nature (cellulose and hemicellulose). In the beginning, a brief overview of each type of biopolymer is provided, focusing on their structure and diversity. An in-depth examination of the latest knowledge related to their design and synthesis methods, formation mechanisms and outstanding properties is provided. A comparative analysis is conducted, focusing on the challenges and advantages of each type of hydrogel. Finally, their biomedical applications are reviewed, focusing on the last five years, briefly highlighting the current challenges of and future development trends in these hydrogels.

## 2. Renewable Biopolymers: Structure and Properties

Hydrogels derived from renewable biopolymers—*cellulose* and *hemicellulose*—have garnered significant attention due to their critical properties, including mechanical reinforcement, biodegradability, water retention capacity and chemical responsiveness. Knowledge of their molecular structure, supramolecular architectures and structural diversity is essential for understanding their interaction mechanisms and their functional roles within three-dimensional (3D) hydrogel networks. These biopolymers exhibit special physicochemical properties, which can be tailored and optimized to meet specific requirements in hydrogel design in order to be used for specific applications.

In this context, the following section presents a brief overview of the structural features and key characteristics of these biopolymers.

**Figure 2:**
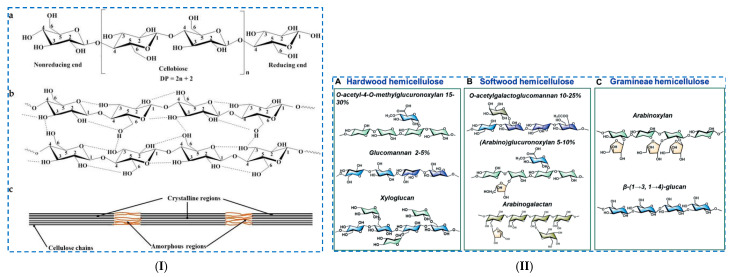
Schematic representation of the following structures: (**I**) Cellulose: (**a**) chemical structure of cellulose, (**b**) hydrogen bonding in cellulose and (**c**) the schematic of cellulose microfibrils. Reproduced with permission from [27]. (**II**) Hemicellulose: (**A**) hardwood, (**B**) softwood and (**C**) Gramineae. Reprinted with permission from [28].

*Cellulose* is a linear polymer, a polysaccharide formed by several D-glucose units (from hundreds to thousands) linked together by β-(1→4) bonds. Each unit has a primary hydroxyl group (OH) at C6 and two secondaries OH groups at C2 and C3, which allow for intra- and intermolecular hydrogen bonding (Figure 2I). Intramolecular hydrogen bonds in cellulose determine its rigid nature, as well as the “double helix axis” of the cellulose molecule, while intermolecular hydrogen bonds determine the laminar nature of cellulose.

Cellulose has a semi-crystalline structure, in which both crystalline and amorphous regions are found. The insolubility of cellulose (in water and most organic solvents) is due to the extended hydrogen bonds established between the macromolecular chains within its crystalline regions, which also contribute to its mechanical strength. This can be overcome either by (i) the use of solvents that chemically react with the OH groups of the cellulose chains, thus destroying the strong interactions between them, especially from the crystalline part of the cellulose, or (ii) the use of solvents that disrupt the established interaction between the OH groups, especially in the crystalline region, and therefore allow the dissolution of the cellulose [29].

Solvent systems, like N-methylmorpholine-N-oxide (NMMO), lithium chloride (LiCl)/ dimethylacetamide (DMAc), dimethylsulfoxide (DMSO)/tertrabutylaluminium fluoride trihydrate (TBAF) and alkali/urea aqueous solutions, have been employed to dissolve cellulose and induce gelation upon regeneration [30,31]. However, there are also special cases when the solvents are either thermally unstable (NMMO), environmentally unfriendly (LiCl/DMAc) or difficult to recycle (TBAF/DMSO) [29,32]. Aqueous solutions of ZnCl_2_ or hydrates of inorganic molten salts have proven to be systems with superior results in dissolving cellulose, and present advantages such as ease of preparation and low cost [33]. Ionic liquids (ILs) have been shown to be more efficient in dissolving cellulose by breaking H-bonds. Among them, better results have been recorded with ILs with aromatic imidazolium cations, which avoid disadvantages such as high solution viscosity and difficulty in dispersing cellulose, but exhibit increased toxicity compared to non-aromatic cations [34]. It should be noted that the length of the alkyl chain is the key factor influencing the toxicity of ILs, and their toxicity increases with increasing alkyl chain lengths. It is important to mention that the development of solvents capable of dissolving cellulose allows the preparation of cellulosic materials in homogeneous systems, eliminating the inconveniences of reactions under heterogeneous conditions, which ensures the involvement of a larger number of hydroxyl groups in the reaction and the achievement of a uniformity of the reaction.

To overcome the limitations of native cellulose and enhance its solubility, cellulose is often chemically modified to produce derivatives, such as carboxymethyl cellulose (CMC), hydroxyethyl cellulose (HEC), methylcellulose (MC), hydroxypropyl methylcellulose (HPMC) and cellulose acetate (CA). These derivatives are soluble in water and common organic solvents and facilitate the formation of hydrogels with tailored properties suitable for specific applications [35,36].

Nanocelluloses (NCs) exhibit remarkable properties, including non-toxicity, biocompatibility, immunogenicity, good mechanical strength and a great reinforcement potential, as well as electrical, thermal and barrier properties, which can be tuned [37]. NCs may be classified into cellulose nanofibrils (CNFs), cellulose nanocrystals (CNCs) and bacterial nanocellulose (BNC). NCs can be extracted from a wide variety of vegetal resources [38,39] and the main extraction processes are mechanical treatment and acid hydrolysis. By varying the lignocellulosic source or the type and severity of the extraction process, a major influence was observed on the size and shape, as well as in morphology and crystalline structure of NCs, which leads to specific and controllable properties. As a result of the unique properties of NCs, such as their large specific surface areas, high modulus and strength, light and strong materials, biodegradability and durability, NC-based hydrogels with greatly improved swelling capacities, mechanical properties and elasticity can be obtained [5]. This fact influences the possibility of using NCs for specific applications, such as high-performance composite materials, packaging, electronics, agriculture and biomedical applications [40,41].

*Hemicelluloses* have a complex structure formed by pyranose and furanose units. These units can be classified into (i) hexoses: D-glucose and D-mannose; (ii) pentoses: D-xylose and L-arabinose; and (iii) acids: galacturonic acid and glucuronic acid. The composition and structure of hemicelluloses vary depending on the plant source, which influences both their properties and the possibilities of creating a hydrogel network (Figure 2II).

Unlike cellulose, which is a homopolysaccharide made up of glucose units, hemicelluloses are heteropolysaccharides composed of various sugar monomers: mannan, xylan, galactomannan, glucomannan, xyloglucan, etc. [42]. Hemicelluloses can be made of (i) unbranched chains ((1-4)-linked xylans or mannans), (ii) helical chains ((1-3)-linked xylans), (iii) branched chains ((1-4)-linked galacto-glucomannans) and (iv) pectin [7]. The backbone contains residues (D-xylose, D-mannose, D-glucose or D-galactose) and other glycosyls as branched chains linked to this chain. The glycosyl groups in hemicelluloses are of several types (pyran type, furan type, α-glycoside bond-linked type, β-glycoside bond-linked type, L- configuration type, D- configuration type, etc.), and the types of linkage between the glycosyl groups vary (1-2, 1-3, 1-4 and 1-6 links). Usually, hemicelluloses exist in the form of branched molecules with a lower molecular weight, which facilitates its chemical modifications and, implicitly, improves its physical and chemical properties.

Hemicellulose can be separated from plant cell walls by breaking down the chemical bonds established with lignin and cellulose. To overcome the problems related to the extraction of hemicelluloses, several methods have been developed, which can be classified into (i) *physical pretreatments* (hydrothermal, steam explosion, microwave irradiation, twin-screw extruder, ultrasonic treatment and subcritical and supercritical fluids), (ii) *chemical pretreatments* (acid extraction, alkaline extraction, organic solvent isolation, ionic liquid extraction and deep eutectic solvent extraction) and (iii) *combined pretreatment* (enzyme–chemical pretreatment, ultrasonic-assisted extraction, microwave-assisted extraction, acid–hydrothermal-assisted process and alkali–hydrothermal-assisted process) [43].

Due to their amorphous nature, hemicelluloses are more soluble in water and alkaline solutions compared to cellulose, which has a crucial impact on their gelation properties, making them an ideal candidate for hydrogel preparation. The structural versatility of hemicelluloses allows for a wide range of possible structures, which can be obtained through different crosslinking mechanisms. However, their low mechanical strength requires either the use of crosslinking agents or their blending with other polymers to form stable hydrogels.

Knowledge of the structural as well as physicochemical properties of cellulose and hemicellulose is absolutely necessary and essential for the design and development of hydrogels with tailored properties. By both capitalizing on the strengths of these biopolymers and addressing and scaling up their limitations, advanced hydrogels can be engineered to meet specific requirements for specific application areas. In this regard, a comparative analysis of the structural characteristics and properties of cellulose and hemicellulose was performed and is presented in Table 1.

## 3. Hydrogel Design and Synthesis Methods

Hydrogels based on cellulose or hemicellulose have attracted particular attention due to their unique structures and physicochemical properties. By choosing certain strategies for preparing hydrogels, specific properties can be tailored, such as improved mechanical strength, high swelling capacity and responsiveness to environmental stimuli.

The preparation of these hydrogels can be achieved using different crosslinking methods:

- *Physical crosslinking*—reversible hydrogel—represents a hydrophilic polymer network made either by physical entanglement of polymer chains or by non-covalent interactions.
- *Chemical crosslinking*—irreversible hydrogel—represents the polymer network made by covalent crosslinking of polymer chains by means of appropriate chemical crosslinking agents.

This section focuses on presenting in detail the current methods used to achieve hydrogels based on the mentioned polysaccharides, highlighting both physical and chemical crosslinking techniques, as well as recent advances in this field.

### 3.1. Physical Crosslinking

**Figure 3:**
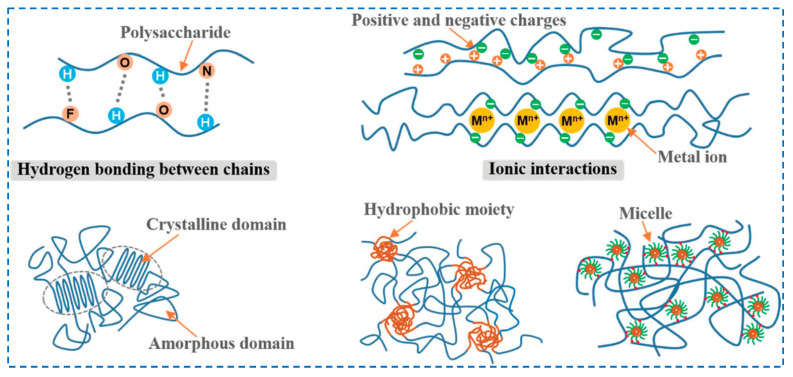
Mechanisms of different physically crosslinked polysaccharide-based hydrogels, such as hydrogen bonding, ionic bonding, phase separation in crystallization, hydrophobic interaction and micellar crosslinking. Reproduced with permission from [47].

Physical crosslinking methods rely on non-covalent interactions, such as hydrogen bonds, crystallization, ionic interactions, hydrophobic interactions, metal coordination, van der Waals forces, entanglement of chains, etc. (Figure 3) [44,45,46].

These physical interactions can be achieved by heating or cooling polymer solutions, freeze–thaw cycles, varying pH and using certain anionic and cationic polymers [47]. The main advantage of this method is that it avoids the use of chemical crosslinking agents (reducing potential cytotoxicity), and moreover, it is a method that is easy to perform and safer. However, hydrogels formed by physical crosslinking may be less stable compared to those formed by chemical methods, and their properties may be more sensitive to changes in temperature or ionic strength [5]. Various methods can be used to produce hydrogels through physical crosslinking reactions, such as freeze–thaw techniques, ultrasound procedures, use of polymerized entanglements, polyelectrolyte complexes (a mixture of polycationic polyelectrolytes with polyanionic polyelectrolytes) or ionic interactions [5,43].

One of the most important non-covalent interactions underlying the formation of hydrogels is *hydrogen bonds*, which can be established either between polymer chains and water molecules [48] or between two polymer chains [49] via (i) the interaction of hydroxyl groups with different functional groups from amide, urea, carboxylic acid, etc., or (ii) their interaction with electron-donating groups (pyridine and imidazole groups). The hydrogen bond is established between a highly negative heteroatom (oxygen, nitrogen, halogen) and a hydrogen atom. This interaction is particularly advantageous because it allows the establishment of an interaction that exceeds the performance of other types of non-covalent bonds, promoting chain flexibility and leading to an increase in mechanical strength [50,51]. These bonds can improve cohesion by acting as crosslinking domains [52] so that the established structure is able to withstand mild mechanical stresses (stretching or bending) applied to the material [53].

*Ionic crosslinking* is characterized by simple reaction conditions and a capacity to crosslink at room temperature. The ionic interactions used in hydrogel formation consist of the physical crosslinking between two molecules with opposite electrical charges [54,55]. Interactions can occur [56] (i) either between a polymer and an oppositely charged small molecule as a linker or (ii) between two oppositely charged polymers. One of the advantages of ionic interaction is its self-healing capacity, a property that can be used in various applications, from aerospace to biomedical applications (tissue regeneration). This consists of breaking the structure of hydrogels under a certain stress and reforming the physical network as soon as the stress is removed [57].

In the physical crosslinking of hydrogels by *crystallization (freeze–thaw)*, the crystallites of the polymer chains act as physical crosslinking sites between macromolecular chains, which, following repeated freeze–thaw cycles, lead to the formation of the 3D polymer network [58]. This process consists of two steps [59]: (i) the freezing step of the polymer solution, when the growth of crystallites is induced, and (ii) the thawing step, in which the polymer chains relax and move freely. By performing several such cycles, the number of crosslinking points increases, leading to a stronger crosslinking of the hydrogel. However, the properties of these hydrogels depend on several factors, such as number of freeze–thaw cycles, the polymers ‘molecular weight, concentration of the aqueous solution, temperature and time. The advantages of this method are that it is simple, easy to perform and non-toxic, yielding hydrogels that can exhibit superior mechanical properties, good biocompatibility and excellent swelling capacity.

The use of *hydrophobic interactions* for crosslinking hydrogels is possible for water-soluble polymers with hydrophobic end groups (i.e., methyl or hydroxypropyl groups) [60,61]. The methods that allow this type of physical crosslinking are (i) thermal induction, based on lower critical solution temperature (LCST) or upper critical solution temperature (UCST) and (ii) ultrasonic treatment. One of the strongest non-covalent interactions is the metal–ligand interaction (*metal coordination*) that is established between metal ions and functional groups in polymer chains. This method is often used in the preparation of hydrogels and can be considered a special Lewis acid–base interaction. It is important to mention that metal–ligand interactions are dynamic, which has a major influence on the self-healing properties of hydrogels and implicitly on the synthesis of stimuli-responsive supramolecular hydrogels [62,63]. The ability of metal-coordination bonds to reform after breaking allows the preparation of hydrogels with tunable, self-organizing and self-repairing mechanical properties [64,65].

#### 3.1.1. Cellulose-Based Hydrogels

Several methods have been developed for the preparation of cellulose-based hydrogels, which differ both in their complexity and in their ability to tailor the desired properties of the final product.

##### Hydrogen Bonding

The abundance of hydroxyl groups in the cellulose structure facilitates the formation of hydrogen bond networks, leading to the formation of a gel and its stabilization in a complex hydrogel structure.

An example in this regard was the preparation of hydrogels based on CMC and phytic acid (PA), an unconventional crosslinking agent, using a one-step method [66]. The introduction of PA into hydrogels, in addition to stabilizing the 3D network of the hydrogels, also endows them with antioxidant and antibacterial properties. The achievement of physical crosslinking in hydrogels was confirmed by FTIR investigations, when it was observed that with an increase in the PA content, the broad band in the 3650–3200 cm^−1^ region, attributed to OH vibrations, decreases and shifts to shorter wavelengths. This is due to the hydrogen bonds established between the OH groups of CMC and PA. Another example of a physically crosslinked hydrogel is one prepared from oxidized hydroxypropyl cellulose (Ox-HPC) and carboxymethyl chitosan (CMCS) through hydrogen bonding and Schiff base reaction [67]. The 3D network of the hydrogel was achieved by simply combining the ketones of the side chains of Ox-HPC with the amines of CMCS in water. Following rheological studies, it was established that the Ox-HPC/CMCS hydrogel was dynamic and self-healing. In addition, the hydrogel with a homogeneous structure was pH sensitive and can be injected precisely, minimizing possible side effects.

**Figure 4:**
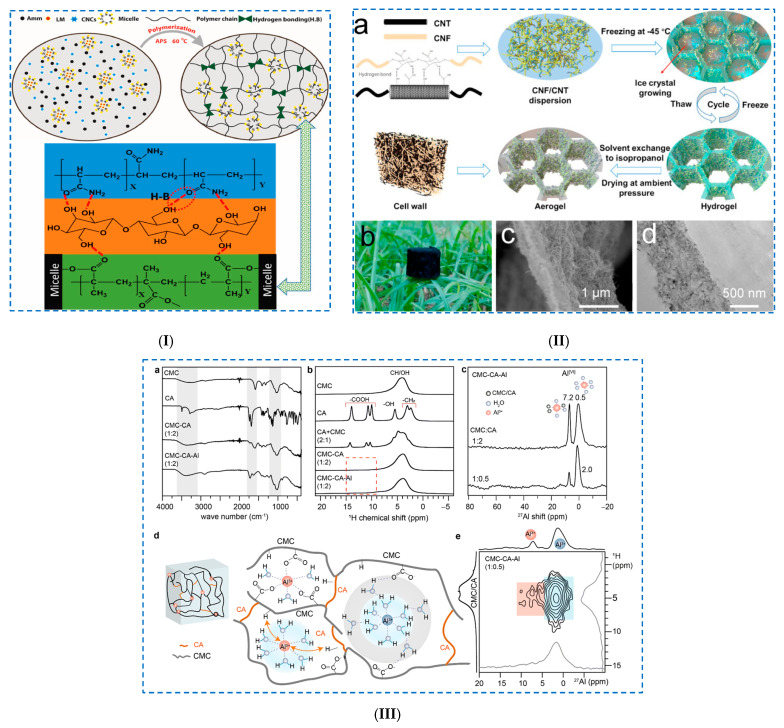
Physical crosslinking of cellulose-based hydrogels: (**I**) Schematic diagram of the polymerization process and the H-bonding of the polymer chains with CNCs. Reproduced with permission from [68]. (**II**) (**a**) Schematic illustration of the synthesis of the CCA composite, (**b**) digital image of the CCA-50 composite and (**c**) SEM and (**d**) TEM image of the pore wall of the CCA-50 composite. Reproduced with permission from [71]. (**III**) (**a**) FTIR spectra of samples, (**b**) solid-state ^1^H MAS NMR spectra of xerogels, (**c**) ^27^Al NMR spectra of CMC–CA–Al, (**d**) schematic of Al^3+^ complexes in close proximity to CMC and CA moieties and (**e**) solid-state 2D ^27^Al–^1^H correlation NMR spectrum plotted along with ^27^Al and ^1^H skyline projections (inset) and the corresponding 1D spectra on the top and left-hand axes. Reproduced with permission from [74].

Khan et al. [68] prepared a dually crosslinked hydrophobic-associated hydrogel (Figure 4I) using (i) *hydrophobic interactions*, where the hydrophobic monomer lauryl methacrylate (LMC) was stabilized in a sodium dodecyl sulfate (SDS) micelle solution. Then, (ii) during the radical polymerization process, these hydrophobic-associated micelles formed partial interactions with the polyacrylamide (PAM) chains. Finally, to include (iii) *hydrogen bonds*, by introducing CNCs, a high number of hydrogen bonds were formed with the polymer chains (dense crosslinking). The H-bonds were established between the OH groups of CNCs and the –NH_2_ and –CO– group of acrylamide (Amm), as well as with the –CO– hydrophilic group of LMC. CNCs were introduced into the hydrogels in order to improve their properties, especially their mechanical properties. It was demonstrated that CNCs conferred exceptional mechanical properties to the hydrogels by establishing new hydrogen bonds, which improved the rigidity of the structure. Due to its high sensitivity (GF = 19.25 at 700% strain), low hysteresis energy (10.9 kJ m^−3^) and high conductivity (22.97 mS/cm), this hydrogel can be recommended as a strain sensor. Furthermore, hydrogels are capable of detecting various human movements (up/down and right/left) of the wrist, fingers and neck, as well as swallowing and speaking.

##### Freeze–Thaw Method

The freeze–thaw technique is a physical method in which cellulose solutions are subjected to repeated cycles of freezing and thawing. These freeze–thaw cycles induce gelation in cellulose solutions by increasing the concentration of polymer, which forces the alignment of macromolecular chains, allowing the association of the chains into 3D networks of hydrogel [69]. Physical hydrogels can also be produced by the repeated freezing and thawing of aqueous solutions of poly(vinyl alcohol) (PVA), a synthetic polymer that is water-soluble, non-toxic, biodegradable and biocompatible. The addition of CNCs in PVA solution and the preparation of hydrogels using the freeze–thaw technique led to an improvement in the compression characteristics of hydrogels and a modification of their 3D structure by decreasing the porosity [70].

Huang et al. [71] prepared a 3D-printable conductive CNF/carbon nanotube (CNT) composite aerogel (Figure 4II). Their study shows that repetitive freeze–thaw cycles cause an entangled of CNTs and CNFs, allowing the production of a hydrogel with superior mechanical properties. Rheological studies have shown that all samples exhibit solid-like behavior (storage modulus, G′ > loss modulus, G″), explained by the establishment of dual network entanglement of nanofibers and hydrogen bonds. Repeated freeze–thaw cycles allowed the formation of increasingly stiff pore walls as the number of cycles increased. Thus, aerogels with tunable densities (0.0519 g·cm^−3^), a large specific surface area (157.24 m^2^/g) and good conductivity (30.95 S·cm^−1^) were prepared.

An anisotropic aerogel was obtained through a physical process of crosslinking CNFs and CNTs using multiple unidirectional freeze–thaw cycles [72]. By rheological measurements, it was demonstrated that the cyclic freeze–thaw processes allowed the formation of nanofiber entanglements, as well as a significant improvement in the mechanical properties of the samples. Thus, the authors managed to create a dual hybrid network of hydrophobic/hydrophilic nanofibers using the monodispersion of CNFs and CNTs. The obtained CNF/CNT aerogel exhibited good mechanical properties (stress < 329.8 kPa at 75% strain), an ultra-low density (0.0262–0.0296 g·cm^−3^) and a high porosity (98.1–98.6%).

##### Ionic Interactions

Shu et al. [73] prepared an ionic hydrogel by dissolving cotton cellulose and PVA in a concentrated ZnCl_2_/CaCl_2_ solution, crosslinked at room temperature. Cellulose-based ionic conductive hydrogels were prepared through (i) a hydrogen bonding crosslinked network, established between cellulose and PVA, and (ii) an ionic interaction crosslinked network, established between Zn^2+^/Ca^2+^ and the OH groups of cellulose. It was established that the Gel-5 hydrogel (0.010 g PVA and 0.2 g cellulose) had the best properties, namely a tensile strength of 0.30 MPa, a compressive strength of 2.05 MPa and a conductivity of 8.16 S m^−1^. All the resulting hydrogels were characterized by high transparency, thermal reversibility and good ionic conductivity, which gave them the possibility of being used as multifunctional sensors for monitoring human movement and temperature.

Thomas et al. [74] synthesized double-crosslinked cellulose-based hydrogels by crosslinking CMC with different crosslinking agents: citric acid (CA), boric acid (BA) and ECH. Of all the crosslinkers, CA was chosen because it allowed soft and stable hydrogels to be obtained. Covalent crosslinking occurs between primary and secondary –OH groups (CMC) and –COOH groups (CA). To further improve the stability of the hydrogel and obtain a dual-crosslinked network, the authors used Al^3+^ ions (Al_2_(SO_4_)_3_·18H_2_O). This multivalent cation bonded with water molecules, forming a stable structure (coordinated aqua-complexes) that either allowed (i) ionic crosslinking with the –COO^–^ groups of CMC and CA or (ii) hydrogen bonds (between –COOH groups of CMC and CA) (Figure 4III). By FTIR spectroscopy, the following was evidenced: (i) the formation of ester bonds between the –OH (CMC) and –COOH (CA) groups (1715–1745 cm^−1^) and (ii) the formation of H-bonds, evidenced by the shift to higher values of the OH stretching vibration (3500–3546 cm^−1^). In addition, the formation of ionic/coordination bonds between the Al^3+^ and –COO^–^ groups of the CMC units were evidenced by a shift to higher values of the stretching vibration of the carbonyl group. The obtained hydrogel films showed not only improved thermal stability but also greater flexibility, ion exchange and pH-sensitive behavior due to the ionic crosslinks established between the CMC, CA and Al^3+^ ions.

Poly(3,4-ethylenedioxythiophene)-coated sulfonated cellulose nanofibers (PEDOT:SCNF) were obtained via a solvent-catalyzed sulfonation process, followed by oxidative self-polymerization and ionic liquid (IL) shielding steps [75]. The PEDOT:SCNF nanofibers were uniformly dispersed within the hydrogel’s network ((PAA) PAA-Al^3+^), prepared from poly(acrylic acid) (PAA), TEMPO-oxidized CNFs (TOCNFs) and Al^3+^ ions. The PEDOT:SCNF nanofibers were obtained from SCNFs (CNFs chemically modified by surface sulfonation) as growth substrates for in situ oxidative polymerization and PEDOT, which spontaneously deposited on the surface of the SCNFs. The ionic liquid Al(TFSI)_3_ was used in the suspension of PEDOT:SCNF to allow for a uniform distribution of the nanofibers and to realize the ionic crosslinking in the hydrogel network. The 3D network of cellulose hydrogels was realized through various physical associations, such as electrostatic interactions, hydrogen bonds, hydrophobic interaction and static π-π stacking. These interactions were due to the presence of the characteristic functional groups of the SCNF, TOCNF and PAA macromolecules within the hydrogels (−SO_3_^−^, −COO^−^, −OH and −CH_2_−). This network gave the hydrogel high stretchability (770%) and super conformability, self-adhesion (28 kPa on pig skin) and self-healing capabilities, which gives it potential applications for electronic skin, for human–machine interfaces or as a healthcare evaluation device.

#### 3.1.2. Hemicellulose-Based Hydrogels

Hemicellulose-based hydrogels can be prepared by several methods, depending on the desired characteristics and targeted applications. These preparation techniques can be broadly categorized into physical crosslinking and chemical crosslinking.

##### Hydrogen Bonding

**Figure 5:**
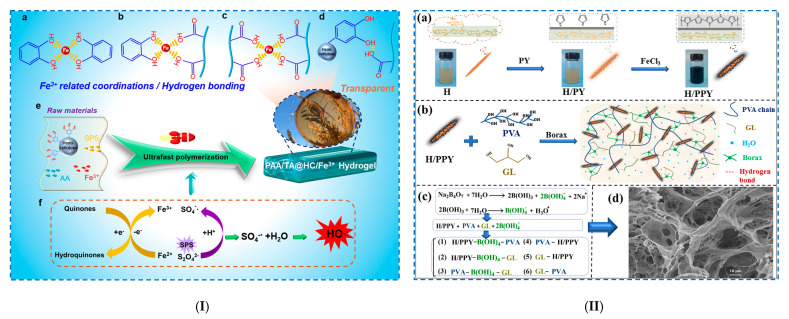
Physical crosslinking of hemicellulose-based hydrogels: (**I**) (**a**–**d**) Different forms of Fe^3+^-related coordination and hydrogen bonding, (**e**) schematic illustration of the polymerization to form PAA/TA@HC/Fe^3+^ hydrogel and (**f**) Fe^3+^/Fe^2+^ and hydroquinone/quinone redox cycle, generating more hydroxyl radicals that are responsible for the fast polymerization process. Reproduced with permission from [76]. (**II**) (**a**) Schematic illustration of the preparation of H/PPY, (**b**) schematic illustration of the synthesis of multifunctional physically crosslinked hydrogels, (**c**) multi-complexation and (**d**) SEM images of the multifunctional physical hydrogel. Reproduced with permission from [78].

Gong et al. [76] made hydrogels from polyacrylic acid (PAA), tannic acid-modified hemicellulose nanoparticles (TA@HC) and Fe^3+^ (Figure 5I). TA@HC is rich in catechol OH groups, which form hydrogen bonds with other hydrophilic groups and reversible interfacial interactions with Fe^3+^ (metal complexes). Furthermore, the addition of TA@HC and Fe^3+^ to the polymerization system with sodium persulfate (SPS) and acrylic acid (AA) decreased the gelation time to 30 s at room temperature (typical radical polymerization processes last > 3 h) by rapidly activating SPS to produce free radicals and generating abundant OH groups in a short period of time. The PAA/TA@HC/Fe^3+^ hydrogel had superior mechanical properties. Thus, for the case of using 0.5 wt% Fe^3+^, a maximum tensile stress of 115 kPa, a maximum deformation of 5600% and a toughness of 4400 kJ/m^3^ were recorded. Furthermore, the hydrogels showed a rapid self-healing ability, good electrical conductivity and reproducible self-adhesion on various substrates (plastic, aluminum, iron, copper, glass, wood and rubber). The adhesion strengths were 8.5 kPa (pigskin), 12.6 kPa (plastic) and 17.8 kPa (paper).

Another example is the preparation of composite hydrogels based on hemicellulose (HC) and graphene oxide (GO) for applications in controlled drug release systems [77]. The HGCH hydrogels were synthesized in a single step, where HC was used as a physical crosslinking agent and GO sheets as a scaffold.

The OH groups on the HC chain could interact with both carboxyl (–COOH) groups and OH groups on the GO surface, establishing a large number of H-bonds between adjacent GO layers, and consequently, promoting gelation. Furthermore, due to the flexible structure of HC, it was possible to establish H-bonds between one chain of HC and two or more GO layers. Thus, an increase in the HC content causes an increase in the number of crosslinking sites, allowing the formation of a stable network.

##### Freeze–Thaw Method

Zhang et al. [78] developed a multifunctional conductive composite hydrogel (PVA/B–GL–H/PPY) via the freeze–thaw method (two cycles), using hemicellulose (H), polypyrrole (PPY), borax (B), glycerol (GL) and PVA (Figure 5II). PVA/B–GL–H/PPY hydrogels were obtained using H as a hydrophilic carrier and PPY as a conductive matrix. PPY alone is unstable and precipitates at the bottom of the flask, but after its deposition on the hemicellulose surface, H-bonds are formed between them, and thus, the H/PPY suspension became much more stable. The formation of the PVA/B–GL–H/PPY hydrogel is explained by the establishment of strong H-bonds between PVA, H/PPY and GL. Borax decomposes in aqueous solutions into borate ions, which form various complexes and crosslinks with H/PPY, GL and PVA. The PVA/B–GL–H/PPY hydrogels exhibited an anti-freeze capacity at extreme temperatures (−20 °C), long-term moisture retention (>7 days) and properties conducive to tensile strain (1094.9%), stress (480.6 kPa), compressive strength (1790.2 kPa) and hydrogel toughness (2.82 MJ/m^3^). The hydrogels also demonstrated excellent durability, presenting 71.8% of their weight after storage for 7 days. It is expected that these hydrogels will be used as epidermal deformation sensors.

##### Ionic Interactions

Tohamy [79] established an environmentally friendly method to prepare a hemicellulose-based hydrogel, which was made of carboxymethyl hemicellulose (CM–Hemi) and nitrogen-doped carbon dots (N–CDs). N–CDs were introduced into hydrogels in order to improve the antibacterial properties of the hydrogels against *Escherichia coli* (Gram-negative bacteria) and *Staphylococcus aureus* (Gram-positive bacteria). The hydrogels were made in the presence of CaCl_2_ with the addition of N–CDs (CM–Hemi@Ca–N–CDs) or without N–CDs (CM–Hemi@Ca). It was established that the CM–Hemi@Ca hydrogel was formed by H-bonds and ionic interactions between the Ca^2+^ and the COO^–^ groups of CM-Hemi chains, which led to a more flexible network and a more elastic structure. The addition of N–CDs to the hydrogel caused strong chemical reactions between Ca^2+^ and CM–Hemi (CM–Hemi@Ca–N–CD hydrogel). Following the condensation reaction, numerous amide bonds (–CO–NH–) were established between the COOH groups on the CM–Hemi surface and the NH_2_ groups on the N–CD surface, which stabilized and stiffened the hydrogel network. Molecular docking studies were performed to demonstrate both antibacterial (against *Escherichia coli* and *Staphylococcus aureus*) and antifungal (*Candida albicans*) properties. The CM–Hemi@Ca–N–CDs hydrogel was shown to exhibit strong binding interactions with the protein of *Staphylococcus aureus* and *Candida albicans* compared to *Escherichia coli*, which confirmed the data obtained by measuring the zone of inhibition in the antibacterial assay. In addition, the establishment of amide bonds between the N-CDs and CM-Hemi led to an improvement in the rigidity of the hydrogel. These results confirm the possibility of using CM–Hemi@Ca–N–CDs as an antibacterial/antifungal sensor.

Table 2 summarizes the latest information on cellulose- and hemicellulose-based hydrogels (2021–2025) and their composition, formation mechanisms and most important characteristics, classified according to physical crosslinking methods.

### 3.2. Chemical Crosslinking

Chemical crosslinking consists of creating irreversible covalent bonds between polymer chains and therefore stable and rigid hydrogels. Generally, this method produces stronger and more stable hydrogels compared to physical crosslinking. Optimizing crosslinking involves striking a balance between achieving a high hydrogel absorption capacity, minimizing the amount of material from a highly absorbent hydrogel that dissolves in the liquid volume and providing adequate mechanical strength.

**Figure 6:**
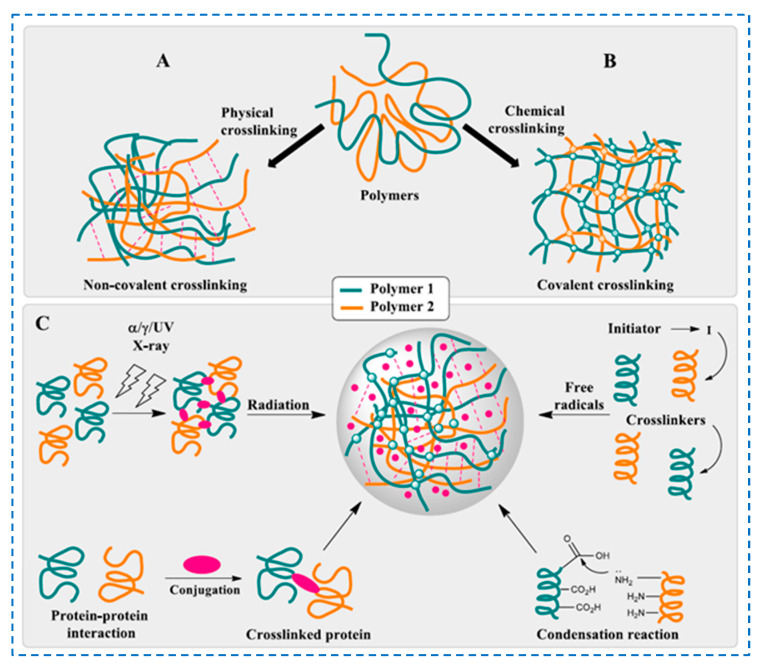
Mechanisms of different crosslinking methods for polysaccharide-based hydrogels: (**A**) physical crosslinking; (**B**) chemical crosslinking; (**C**) various routes of chemical crosslinking. Reproduced with permission from [83].

Different strategies to obtain hydrogels have been reported, and these methods can be classified into [80,81,82,83,84] the following: (i) reactions between complementary functional groups, such as formation of Schiff base, Michael addition, condensation, etc., and (ii) protein/enzyme-mediated crosslinking, (iii) free radical polymerization and (iv) high-energy irradiation (Figure 6).

The formation of chemically crosslinked bonds can occur either in the presence of small crosslinking molecules, or by polymer–polymer conjugation, or by the addition of photosensitizing agents, etc.

*Small molecule crosslinking* agents are molecules with at least two reactive functional groups that are commonly used to form covalent bonds between the macromolecular chains of the polysaccharide (celluloses and hemicelluloses). There are various crosslinking agents, such as epichlorohydrin, ethylene glycol diglycidyl ether, diethylenetriaminepentaacetic dianhydride, sodium trimetaphosphate and polycarboxylic acids (i.e., citric acid, boric acid, itaconic acid, maleic acid, etc.) [85]. For the case of cellulose hydrogels, chemical crosslinking agents can be classified into the following categories [86,87]: (i) *esterification agents*: multifunctional carboxylic acid and carboxylic anhydride, which form –COOR bonds and some peptide bonds (–CONH–) in hydrogels; and (ii) *etherification agents*: organochlorines, epoxides and vinyl compounds, which form R–O–R and some secondary amine bonds (R–NH–R) in hydrogels. These crosslinking agents react with the functional groups of the macromolecular chains and establish new crosslinks between the polymer chains.

*Radiation crosslinking* is an environmentally friendly method for preparing hydrogels, and can be achieved by (i) ultraviolet (UV) irradiation or (ii) high-energy radiation, such as gamma irradiation and electron beams [88,89,90]. Some of the advantages of this method are that it does not contain additives, it is a controllable and rapid gelation and both gel formation and sterilization are achieved simultaneously. The reaction consists of the production of radicals on macromolecular chains under the action of radiation, which either (i) initiate the polymerization of unsaturated bonds or (ii) convert inert groups into active groups.

*Free radical polymerization* is a method in which polymer chains are produced by successive free radical reactions and different types of initiators are used to generate free radicals. These initiators could be thermal, redox or photoinitiators (UV radiation and electron beams). The formation of three-dimensional hydrogel networks can be achieved by various polymerization mechanisms, including step growth, chain growth or a combination of these two types of polymerization [91,92,93].

*Click chemistry reactions* are simple and rapid reactions with numerous advantages (i.e., high yields under mild conditions, fewer by-products and high specificity and selectivity) [94,95]. Some of the classical crosslinking methods by “click chemistry” are Schiff base, Diels–Alder, Michael-type addition, etc. [96,97]. Among them, copper-catalyzed azide–alkyne cycloaddition and thiol-ene/yne click reactions are the most commonly used for polymer modification. Furthermore, the thiol-ene reaction can be carried out under mild reaction conditions with high conversion and selectivity and without the use of toxic metal catalysts [82].

*Schiff bases* are organic compounds that contain in their structure, for example, azomethine or imine groups, and can be obtained from the condensation reaction of a carbonyl group with an amine group [98,99]. The Schiff reaction is an effective method for the preparation of hydrogels based on cellulose or hemicellulose: (i) these biopolymers are first chemically modified by introducing reactive functional groups, an aldehyde group (–CHO) or a ketone group (–C=O); then, (ii) the modified polymers are mixed with compounds containing an amino group (such as ethylenediamine, 1,2-phenylenediamine, etc.); and finally, (iii) under specific pH and temperature conditions, the amine compounds react with the reactive carbonyl groups of biopolymers, forming a Schiff base-crosslinked structure. There is a dynamic equilibrium between the Schiff base bonds and the aldehyde and amine reactants (pseudo-covalent bonds), which by uncoupling/recoupling the imine bonds in the hydrogels, imparts their self-repair capacity [100].

One of the most recent methods for preparing in situ hydrogels is *enzymatic crosslinking*, which is based on a crosslinking reaction catalyzed by various enzymes (such as transglutaminases, peroxidases, tyrosinase, phosphatases, etc.) [35,101,102].

#### 3.2.1. Cellulose-Based Hydrogels

##### Ring-Opening Reactions

One of the most widely used crosslinking agents in the preparation of cellulose-based hydrogels is epichlorohydrin (ECH). Usually, the crosslinking reaction takes place in a basic environment, in the presence of a cheap and readily available solvent (e.g., 6–9% NaOH solution) and without the need for a catalyst. The crosslinking reaction occurs between the epoxy group of ECH and the OH group of cellulose through a ring-opening step of the epoxy group, followed by an addition reaction with other functional groups to form a crosslinked structure. The secondary compounds of the reaction are NaCl and possibly unreacted ECH, which can, however, be completely removed from the hydrogel via repeated washings. It should be noted that in biomedical applications, epoxy compounds are preferred over, for example, dialdehydes or divinyl sulfones because are less toxic [103].

**Figure 7:**
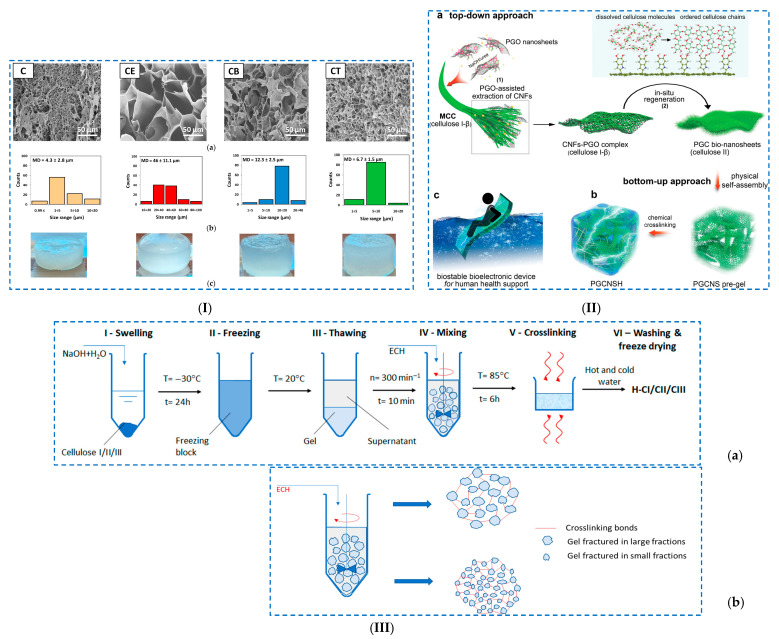
Chemical crosslinking of cellulose-based hydrogels—*ring-opening reactions*: (**I**) (**a**) Cross-sectional micrographs (mag 500×), (**b**) pore size distribution histogram (MD—mean pore diameter) and (**c**) photographs of never-dried hydrogels. Reproduced with permission from [103]. (**II**) Design strategy for 2D conductive cellulose nanosheets and their assembly into biostable and conductive 3D hydrogels. (**a**) Top–down approach for the formation of PGC bio-nanosheets, (**b**) bottom–up approach for the formation of PGC bio-nanosheet-assembled hydrogel (PGCNSH) and (**c**) conductive and biostable PGCNSH as a bioelectronic device to support human health. Reproduced with permission from [104]. (**III**) (**a**) The schematic method for the preparation of hydrogels based on cellulose allomorphs, and (**b**) a model for hydrogel synthesis. Reproduced with permission from [105].

An example of a chemical crosslinking reaction in the presence of ECH is the case of hybrid cellulose bio-nanosheets (PGC) with dopamine-reduced graphene oxide (PGO) [104] (Figure 7I).

The mechanism of PGC bio-nanosheet hydrogel formation consists of the following: (i) cellulose is dissolved in a NaOH/urea system, and PGO nanosheets are introduced as a supporting template to guide the arrangement of cellulose molecules in a CNF-PGO complex; this complex is formed by hydrogen bonds established between the PGO nanosheets, with numerous catechol groups and the OH groups of CNFs; (ii) then, the CNF-PGO complex is subjected to freeze–thaw cycles, which allows the dissolution of the CNFs and their in situ regeneration as cellulose II on the PGO surfaces, with the formation of PGC bio-nanosheets; (iii) a PGC bio-nanosheet-assembled hydrogel (PGCNSH) is achieved via a combined process of (a) self-assembly of PGC bio-nanosheets through non-covalent interactions, which (b) are crosslinked in the presence of ECH (chemical crosslinking). The formation mechanism of the 3D network was obtained through XRD analysis and FTIR spectroscopy studies.

Nicu et al. [103] obtained hydrogels based on cellulose by using different crosslinking agents from the same family (glycidyl family), such as (i) ECH, (ii) 1,4-butanediol diglycidyl ether (BDDE) and (iii) trimethylolpropane triglycidyl ether (TMPTGE). The obtained hydrogels showed significant differences in terms of network morphology, pore size distribution and gel fraction values, which had an important influence on the swelling degree and rheological and thermal properties (Figure 7II). Given that the degree of swelling (Qt) of hydrogels follows the series Qt_CE_ > Qt_CB_ > Qt_CT_, it was demonstrated that the more the number of epoxy groups in the crosslinking agent structure increases, the more compact 3D networks are formed. These hydrogels have proven exceptional rheological properties, especially with regard to the elastic component. Ciolacu et al. [105] synthesized hydrogels starting from three different allomorph forms of cellulose (CI, CII and CIII) by treating cellulose in a NaOH aqueous solution, followed by chemical crosslinking with ECH (Figure 7III). It was observed that the gelation stage in the synthesis of hydrogels plays a key role in obtaining hydrogels with different performances and swelling of cellulose allomorphs in NaOH solutions at low temperature (−30 °C), leading to the attainment of gels with different strengths and rheological characteristics. The most compact gel was recorded for CII, a gel that was more difficult to fragment, and the least dense and easiest to fragment was found in the case of CIII. As a consequence, less crosslinked hydrogels (H–CII) could be prepared, which have a high swelling capacity, while the H–CIII hydrogels showed lower swelling capacities, even compared to those obtained from native cellulose (H–CI), but had superior rheological and strength properties (Q_H–CII_ > Q_H–CI_ > Q_H–CIII_).

Another example concerns the preparation of a hydrogel from cellulose fibers derived from waste paper and CMC, which were crosslinked in the presence of ECH at low temperature (at −20 °C) [106]. Among all the formulations, the C3 hydrogel (3% (*w*/*v*) cellulose and 1.75% (*w*/*v*) CMC) exhibited (i) a high swelling capacity (2000%), which resulted in improved soil moisture retention, and (ii) a controlled release of fertilizers, demonstrating that it could be used in agricultural applications.

##### Polymerization Reactions

**Figure 8:**
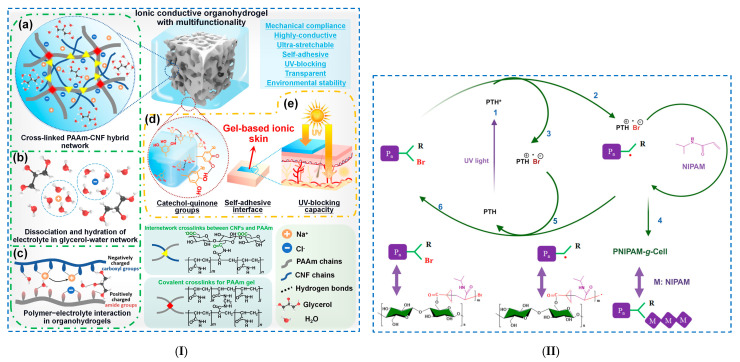
Chemical crosslinking of cellulose-based hydrogels—*polymerization reactions*: (**I**) (**a**) The formation of a PAAm-CNF interpenetrating network and aqueous electrolyte (Na+, Cl− and glycerol–water binary solvent) in the organohydrogel, (**b**) the dissociation and hydration of electrolytes in the glycerol–water network, (**c**) the interaction between the PAAM-CNF hybrid skeleton and electrolyte in the organohydrogel, (**d**) the catechol–quinone groups formed on the surface of the organohydrogel and (**e**) the formation of gel-based ionic skin. Reproduced with permission from [107]. (**II**) The synthesis mechanism of PNIPAM–g–Cell involves the following steps: (1) UV light excites PTH to form the excited-state PTH*, (2) the initiator reacts with the excited-state PTH*, (3) PTH* transfers electrons to Br, (4) the excited-state polymer initiator reacts with monomer NIPAM, (5) PTH returns to its ground state and (6) PTH*+ is deactivated. Reproduced with permission from [109].

The photoinitiated crosslinking reaction requires the presence of photoinitiators to initiate the radical polymerization reaction in which the monomers and the crosslinking agent react to form a 3D network structure. Wei et al. [107] made organohydrogel-based ionic skins via a UV-initiated polymerization process (Figure 8I). The hydrogels were prepared from a polyacrylamide (PAAm)/CNF network, functionalized with tannic acid (TA), where electrolytes (NaCl) and a glycerol–water binary solvent were introduced. CNFs were used to improve the mechanical properties of the hydrogel, while tannic acid was used to improve the adhesion and UV-blocking capacity of the hydrogel. The mixture was chemically crosslinked under UV irradiation by free radical polymerization. The mechanism of hydrogel network formation consisted of several steps: (i) PAAm: covalent crosslinking of acrylamide monomers in the presence of the crosslinker *N*,*N*′-methylenebisacrylamide (MBA) and the initiator (2,2′-azobis [2-(2-imidazolin-2-yl)propane] dihydrochloride (AIBI); (ii) PAAm/CNF: CNFs form interactive entanglements with PAAm chains; (iii) addition of NaCl: induces ionic conductivity of the organohydrogel; and (iv) introduction of a binary solvent system of glycerol–water: easy formation of supramolecular interactions (the hydrogen bonds established between glycerol and water molecules reduce moisture evaporation in an open environment and prevent the formation of ice crystals at low temperatures). The hydrogels presented superior properties, such as improved stretchability (<1430%), good Young’s modulus (23 kPa) and ionic conductivity (2.7 S/m).

Hydrogels based on cellulose and poly(dimethylaminoethyl methacrylate) (PDMAEMA) were prepared by copolymerization of *N,N*-dimethylaminoethyl methacrylate (DMAEMA) in cellulose solution [108]. The polymerization of DMAEMA in cellulose solution makes it possible to produce several compounds, such as (i) a copolymer with a branched structure, (ii) a microgel, (iii) semi-interpenetrated networks and (iv) a compound in which the polymer chain is grafted onto the cellulose chain. In addition, irradiation of the PDMAEMA–cellulose mixture (by gamma irradiation) was also chosen to achieve complete grafting/crosslinking of the polymers. The authors studied both the influence of the cellulose/DMAEMA molar ratio and the irradiation dose (10, 30 or 100 kGy) on the conversion of the polymerization reaction and crosslinking efficiency. It was observed that by increasing the irradiation dose of the samples, an increase in the efficiency of the grafting/crosslinking reaction of the material was obtained. The achievement of the grafting reaction of PDMAEMA molecules on the cellulose chain was demonstrated by FTIR spectroscopy. The PDMAEMA content in the 3D structure of the hydrogels had a major influence on the degree of their swelling and reswelling and their ability to produce Ag particles, as well as to absorb Fe^3+^ ions, facts which recommend them as possible hydrogels in wound treatment.

Liu et al. [109] synthesized a thermosensitive, drug-loaded injectable hydrogel (PNIPAM-g-Cell) using 2-bromoisobuturyl bromide-modified cellulose as the macroinitiator and 10-phenylphenothiazine (PTH) as the catalyst under UV light (Figure 8II). A metal-free photoinduced atom transfer radical polymerization (ATRP) reaction was used to prepare the thermosensitive cellulose graft copolymer. The polymerization of N-isopropylacrylamide (NIPAM) was UV-activated with an organic-based photoredox catalyst. The confirmation of the reaction’s success was achieved by FTIR, ^1^H NMR and ^13^C NMR spectroscopic investigations. It was observed that at concentrations higher than 5% in water, PNIPAM–g–Cell can form a thermosensitive, micelle-like injectable hydrogel (37 °C) with good biocompatibility.

##### Schiff Base Reaction

A composite hydrogel was prepared based on oxidized hydroxyethylcellulose (OHEC) and allyl copolymers [110]. In this case, the molecular chains of OHEC served as macromolecular templates for the formation of Schiff bases, boron–oxygen and hydrogen bonds in the scaffold. The poly (hydroxyethyl acrylate-co-acrylic amide) (P(HEA-co-AAm)) hydrogel was fabricated using free radical polymerization from OHEC, AAM and 2-Hydroxyethyl acrylate (HEA) with double bond-functionalized spirooxazine (DSPO) (2-Propenoic acid, 2-methyl-2-[(1,3-dihydro-1,3,3-trimethylspiro-[2H-indole-2,3′-[3H]-naphtha[2,1-b][1,4]oxazin]-9′-yl)oxy]ethyl ester). By FTIR spectroscopy, the establishment of bonds in the P(HEA-co-AAm)-OHECDSPO hydrogel was identified: (i) the OH and NH groups were identified to be involved in hydrogen bonding, (ii) Schiff base bonds were formed between the -CHO (OHEC) and -NH_2_ groups of the macromolecular chain of (P(HEA-co-AAm)) and (iii) boron–oxygen bridges were established between borax and HEA. These hydrogels demonstrated superior mechanical properties, good pH sensitivity and non-cytotoxicity in vitro, with potential applications as smart contact lenses.

A dual network (DN) hydrogel was made from cellulose and chitin, with the idea of using it as a multifunctional conductive strain sensor [111]. The first layer of the hydrogel was prepared by a Schiff reaction between dialdehyde carboxymethyl cellulose and amino gelatin, while the second layer of the network was formed by free radical polymerization of acrylic acid (AA). The biocompatible hydrogel thus obtained was shown to be compressible up to 0.12 MPa (when compressed to 50% of its original height). It also exhibited superior cyclic compression properties, with a minimum of 10,000 cycles, and excellent electrical conductivity. In addition, the hydrogel exhibited excellent fatigue resistance and cyclic adhesion properties, with the adhesion strength remaining unchanged after 10 cycles. Yin et al. [112] used the Schiff base reaction to construct composite self-healing hydrogels. In this regard, a crosslinking reaction was carried out between the aldehyde group of oxidized microcrystalline cellulose (OMCC) and the amino group of CMCS. The prepared hydrogels exhibited an adequate gelation time (minimum 54 s), a distinct swelling rate (approximately 31.18 g/g), good mechanical properties and a good coagulation effect. Furthermore, it was demonstrated that the release of rutin could be controlled by the degree of crosslinking between the modified OMCC and CMCS based on the Schiff base reaction.

#### 3.2.2. Hemicellulose-Based Hydrogels

##### Ring-Opening Reactions

A composite hydrogel based on corn cob hemicellulose (CHC) and chitosan (CS) was produced through a two-step process [113]: (i) dissolving CHC and CS in an alkali/urea/water system using the freeze–thaw method (in three cycles) and (ii) crosslinking CHC and CS with ECH. FTIR spectroscopy investigations showed that a chemical crosslinking reaction in the presence of ECH took place. SEM microscopy showed that the HC/CS hydrogel had a honeycomb structure, with a uniform appearance and a pore size of approximately 200 μm. However, an increase in the CS content (from 1% to 5%) caused a decrease in the swelling ratio of the hydrogel (from 22.59 to 6.62) and an increase in the compressive strength (from 0.104 MPa to 0.325 MPa).

##### Small Molecule Chemical Crosslinking

Hydrogels from beech xylan were synthesized through a covalent crosslinking reaction of xylan in the presence of diethylenetriaminepentaacetic dianhydride (DTPA) [114]. It was confirmed by FTIR spectroscopy that an increase in the amount of DTPA dianhydride in the system increased not only the degree of crosslinking of the hydrogel but also the hydrophilicity of the hydrogel by introducing three new carboxyl groups. The hydrogel exhibited a high swelling ratio (<62), a macroporous honeycomb structure and good mechanical properties.

**Figure 9:**
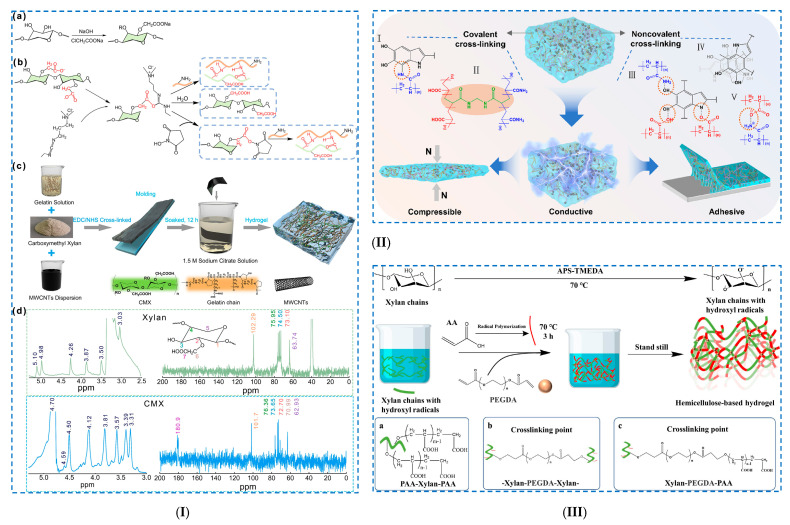
Chemical crosslinking of hemicellulose-based hydrogels: (**I**) Preparation of xylan-based hydrogels: (**a**) synthesis of CMX, (**b**) formation of stable amide bonds by crosslinking CMX with gelatin via EDC/NHS-system, (**c**) schematic illustration of the synthesis of CMX hydrogels, (**d**) ^1^H NMR spectra and ^13^C NMR spectra of xylan and CMX. Reproduced with permission from [115]. (**II**) Schematic diagram of the conductive, self-adhesive hydrogel. Reproduced with permission from [121]. (**III**) Insights into XH-Gel hydrogel formation. Reproduced with permission from [123].

Zhu et al. [115] developed a hydrogel based on carboxymethyl xylan (CMX) and gelatin (G), doped with conductive hydroxyl carbon nanotubes (OCNTs) (Figure 9I). A semi-interpenetrating hydrogel network was established between CMX and G, containing amide bonds and numerous intermolecular hydrogen bonds.

The covalent amide bonds were established between CMX (by the –COOH groups) and G (by the –NH_2_ groups), using 1–(3–dimethylaminopropyl)–3–ethyl carbodiimide hydrochloride (EDC) as a crosslinking agent. In addition, the hydrogel was further subjected to a salting-out treatment in the presence of a highly concentrated solution of sodium citrate dihydrate (Na3cit), which led to the aggregation of molecular chains and to the formation of hydrophobic interaction regions in the hydrogel network. Moreover, the application of each successive treatment, such as (i) chemical crosslinking, (ii) salting-out treatment and (iii) doping of hydrogels with OCNTs, allowed the attainment of composite hydrogels with increasingly better mechanical properties. The CMX1-g-G3/OCNT composite hydrogel presented superior mechanical properties (tensile stress of 1.63 MPa and compressive stress of 1.5 MPa).

##### Polymerization Reactions

Liu et al. [116] prepared nanocomposite hydrogels from dopamine-grafted carboxymethyl xylan (CMX–DA) that was crosslinked with polyacrylamide (PAM), and bentonite was introduced as a nano-reinforcing material. DA was introduced to give the hydrogel adhesion properties, while bentonite was introduced to increase the mechanical properties of the hydrogel. The mechanism proposed by the authors for the synthesis of hydrogels consisted of several consecutive reactions, such as (i) AA polymerization into PAM in the presence of ammonium persulfate (APS) and MBA, (ii) the generation of free radicals in CMX–DA by capturing hydrogen atoms from the –OH groups of CMX–DA by APS and (iii) the grafting of PAM onto the CMX chains. Regarding bentonite, physical interactions were established between it and the polymer chains. The obtained nanocomposite hydrogel demonstrated improved mechanical properties, such as in compressive stress (218.29 kPa), tensile stress (42.17 kPa) and elongation at break (436%), but also good adhesion properties towards glass, plastic, metal and PTFE.

A chemically crosslinked hydrogel was obtained by free radical polymerization of acylated xylan and silanized graphene oxide for possible uses in wastewater treatment and metal ion collection [117]. Xylan was isolated from corn straw and was modified with maleic anhydride (xylan–MAH), while graphene oxide (GO) was modified with vinyltriethoxysilane (GO–VTEO). These modifications were made to introduce into the materials the double bond C=C. The chemically crosslinked composite hydrogels were prepared by free radical polymerization, and their morphology, determined by SEM microscopy, showed that they had a porous honeycomb structure. GO–VTEO–xylan–MAH hydrogels were shown to be pH-sensitive, with the swelling degree being minimal at acidic pH and increasing with increasing pH (>8). One explanation is that with an increase in pH above the pK value of the carboxyl groups (pKa = 4.28), the –COOH groups dissociate into –COO^−^, causing an increase in the electrostatic repulsion force in the hydrogel network and, respectively, an increase in the number of water molecules that can be absorbed by the hydrogel, which causes an increase in the swelling capacity of the hydrogel.

Another example is the preparation of a porous hemicellulose–chitosan–iron ion (HC-CSN-Fe^3+^) hydrogel [118]. This hydrogel was prepared by grafting the molecular chains of HC onto acrylic acid (AA) by free radical copolymerization. In addition, more stable networks are created by chelation with metal ions, more precisely, by complexing Fe^3+^ with the amino groups of CSN, in the HC–CSN–Fe^3+^ hydrogel. The successful introduction of Fe^3+^ into the HC-based hydrogel was evidenced by FTIR spectroscopy. The obtained hydrogels showed improved mechanical properties and UV resistance. Rodríguez-Ramírez et al. [119] synthesized hydrogels based on hemicellulose (HC) and crosslinked polyacrylamide. The hydrogels were prepared by graft copolymerization of HC and acrylamide (Am) in the presence of N,N–methylenebisacrylamide (BIS) (crosslinking agent) and ammonium persulfate/N,N,N′,N′–tetramethylethylenediamine (APS/TMEDA) (a redox initiator system). Bentonite (BT) was added to the system to increase the adsorption capacity of the hydrogels. It was observed that the introduction of BT into the hydrogel made it more rigid due to the fact that BT provides numerous attachment points with the hemicellulose matrix and, implicitly, the formation of new ester bonds. From SEM microscopy, it was observed that the presence of HC in the hydrogel causes a reduction in the porosity of the matrix, explained by the generation of a greater number of hydrogen bonds, which favors its crosslinking in the semi-IPN network. As a conclusion, it was established that in the HC–g–Am–BIS–BT hydrogel, HC acts as a multifunctional crosslinking agent, both through covalent bonds and hydrogen bonds between HC and Am–BIS–BT.

New xylan-based hydrogels were developed via a two-step method [120]: (i) synthesis of dehydroabietic acid (DAGMA) derivative 2–methacryloyloxyisopropanol ester and (ii) copolymerization of xylan, acrylamide (AM) and hydrophobic DAGMA. For this, hydrophobic rosin derivatives were used as physical crosslinking points, APS as the initiator and TMEDA as an accelerator in the copolymerization process of xylan with AM. The size of the nanomicelles was approximately 5 nm, determined by dynamic light scattering (DLS) measurements. FTIR spectroscopy investigations confirmed the following: (i) the copolymerization process of the hydrogels by identification of bands corresponding to the ester group (CO double bond, 1652 cm^−1^) and the aromatic ring (1495 cm^−1^), and (ii) the formation of the hydrogen bonds between xylan and PAM polymer chains. Xylan-based hydrogels were shown to have good mechanical properties, such as a tensile strength of 0.34 MPa and a toughness of 3.79 ± 0.95 MJ/m^3^. However, by adding MXenes as conductive fillers, superior mechanical properties were obtained (strength of 0.51 MPa and toughness of 5.95 ± 1.19 MJ/m^3^).

Li et al. [121] obtained hemicellulose-based hydrogels reinforced with nano-polydopamine (ND) in order to create flexible materials for use in health monitoring and self-administration (Figure 9II). HC-based hydrogels (P(AM–AC)–HC–NP) were prepared by (i) crosslinking HC with nano-polydopamine through amination reactions and (ii) copolymerizing HC/ND with acrylamide (AM) and acrylic acid (AC). The obtained ND particles showed storage stability and a dual effect of chemical and physical crosslinking (through hydrogen bonds and electrostatic interactions). The H-bonds between ND and the polymer matrix occurred mainly between the catechol group (NP) and amino groups (PAM) and carboxyl groups (PAC). In the copolymerization reaction, the –OH groups of HC are oxidized to oxygen radicals, which function as efficient grafting sites for AM or AC. Moreover, strong covalent bonds are formed in the presence of MBA, established between the catechol groups (ND) and amino groups (PAM), leading to the formation of an interpenetrating network. The nanocomposite hydrogels exhibit excellent mechanical properties (maximum compressive strain of 88% and compressive stress of 650 kPa), and after 1000 cycles of cyclic compression, do not show clear signs of crushing. In addition, the P(AM–AC)–HC–NP hydrogels exhibit good electrical conductivity and self-adhesive properties. Shen et al. [122] prepared a “smart” composite hydrogel through a double network formed by alginate/Ca2+ and polyacrylic acid-co-dimethylaminoethyl methacrylate [P(AA-co-DMAEMA)] in which hemicellulose-based nanoaggregates were incorporated for the aim of being used as a controlled drug release system. Two types of hemicellulose-based nanoaggregates were obtained: (i) nanoaggregates of xylan-rich hemicellulose laurate polymers (XH–LA–MA) chemically incorporated into the hydrogel and (ii) nanoaggregates of hemicellulose laurate (XH–LA) physically incorporated into the hydrogel. Hydrophobic modification of vinyl-functionalized hemicellulose (XH–LA–MA) was achieved by (i) esterification of xylan-rich hemicellulose with lauric acid (LA) and (ii) a subsequent reaction with glycidyl methacrylate (GMA) via transesterification. Hemicellulose-based nanoaggregates were mixed in hydrogels made of (i) an ionically crosslinked sodium alginate/Ca^2+^ network and (ii) a P(AA–co–DMEMA) network crosslinked by free radical polymerization. XH–LA–MA nanoaggregates participated in the crosslinking of the hydrogel through (i) covalent bonds (via vinyl groups), but also through (ii) H-bonds between the hydroxyl groups of hemicellulose and the amino and carboxyl groups of P(AA–co–DMEMA) and sodium alginate.

Long et al. [123] developed a dual pH/magnetic response nanocomposite hydrogel based on xylan hemicellulose (XH) to be used in drug delivery (acetylsalicylic acid and theophylline) (Figure 9III). To obtain the XH-based hydrogel, poly(ethylene glycol) diacrylate (PEGDA) was used as a crosslinking agent, and Fe_3_O_4_ magnetic nanoparticles (Fe_3_O_4_ MNPs) were incorporated into the matrix to induce a dual pH/magnetic response. A more complex 3D structure of the Fe_3_O_4_@XH–Gel hydrogel was prepared through several steps: (i) generation of free radicals on XH chains by capturing hydrogen atoms from OH groups and grafting acrylic acid (AA) onto XH chains (PAA–Xylan–PAA), (ii) free radical polymerization of polyacrylic acid (PAA), (iii) crosslinking reaction in the presence of PEGDA (Xylan–PEGDA–Xylan) and (iv) crosslinking reactions between PEGDA and PAA (Xylan–PEGDA–PAA). The successful grafting and crosslinking of AA onto XH, as well as the encapsulation of Fe_3_O_4_ MNPs in the 3D network, was confirmed by FTIR and ^1^H NMR spectroscopies. It was demonstrated that at a pH of 1.5 (gastric juice), the hydrogel has a limited swelling capacity, and the drug, a low release capacity. At a pH of 7.4 (intestinal tract), the hydrogel exhibits a maximum swelling capacity and a maximum release capacity. Thus, it was demonstrated that the hydrogel exhibits pH-dependent behavior, which allows targeted drug release, especially in gastrointestinal diseases.

##### Schiff Base Reaction

Guo et al. [124] synthesized hemicellulose-based hydrogels with superior mechanical properties, strengthened by the Hofmeister effect. The authors selected the ions of SO_4_^2−^, S_2_O_3_^2−^, H_2_PO_4_^−^, CO_3_^2−^, CH_3_COO^−^, Cl^−^, Li^+^, Na^+^, K^+^, Ba^2+^, Ca^2+^ and Fe^3+^ from the Hofmeister series and studied the effects of the ions on the structure and properties of hemicellulose-based hydrogels. Conductive composite hydrogels with a double-crosslinked physicochemical network were prepared from dialdehyde xylan (DAX), gelatin and polyvinyl alcohol (PVOH). The 3D network of the PVOH/gelatin-DAX hydrogel (I–PGD) consisted of (i) a chemically crosslinked network through the Schiff base reaction, established between DAX and gelatin, and (ii) a physically crosslinked network of PVOH, sensitive to saline solution. Then, the I-PGD composite hydrogel was immersed in a Na_2_SO_4_ solution to obtain a Hofmeister-enhanced conductive composite hydrogel (H–PGD). It was observed that under the influence of SO_4_^2−^, the hydrogel shrinks considerably due to the entanglement of the PVOH molecular chains, which leads to the formation of a denser network. It was determined that the I–GD hydrogel exhibits poor mechanical properties at a deformation of 80%: tensile strength of 29.4 kPa, elongation of 80.3% and a compressive strength of 0.57 MP. By applying the Hofmeister effect, the mechanical properties of hemicellulose-based hydrogels were substantially improved due to the induction of polymer chain aggregation by ions during salinization. Comparing the mechanical properties of the hydrogel, with Na^+^ as a constant counterion, the Hofmeister series was established: SO_4_^2−^ > S_2_O_3_^2−^ > H_2_PO_4_^−^ > CO_3_^2−^ > CH_3_COO^−^ > Cl^−^. After immersing the hydrogel in the Na_2_SO_4_ solution, a significant increase in mechanical properties was observed: tensile strength of 3.02 MPa, elongation of 330.95% and tensile modulus of 1.79 MPa.

##### Thiol-Ene Reaction

Wang et al. [125] produced an injectable nanocomposite hydrogel through the thiol-ene crosslinking reaction between methacrylate-modified O–acetyl–galactoglucomannan (GGMMA) and thiol-grafted cellulose nanocrystals (CNCs–SH), used in various biomedical applications, from wound dressing to TE scaffolds. The thiol-ene crosslinking reaction involves an orthogonal increase in the addition of thiol-ene, which allows the formation of a more uniform hydrogel network with a faster crosslinking rate. The interpenetrating network obtained by the addition of thiol-ene is achieved by the action of CNCs–SH (i) either as a nanofiller, reinforcing the GGMMA hydrogel, (ii) or as a crosslinking agent towards GGMMA. Thus, the mechanical properties of the GGMMA/CNCs-SH hydrogel are strongly influenced by the ratio between the thiol groups (CNCs–SH) and the methacrylate groups (GGMMA). Furthermore, the obtained hydrogels were loaded with bioactive glass nanoparticles (BaGNPs) and tested as delivery systems for therapeutic ions (Si, Ca and Cu ions) in simulated body fluid. It was demonstrated that the hydrogels exhibit the ability to release therapeutic ions in a sustained manner (up to 14 days), which recommends them for use in medical applications.

Table 3 summarizes the latest information on cellulose- and hemicellulose-based hydrogels (2021–2025), including their composition, formation mechanisms and most important characteristics, classified according to chemical crosslinking methods.

## 4. Hydrogels’ Performances

### 4.1. Swelling Behavior

The swelling of hydrogels is a key characteristic regarding the functionality of hydrogels in different environments and their ability to retain water while maintaining a certain structural integrity. There is an interconnection between the chemical structure (presence of hydrophilic and hydrophobic groups), the degree of crosslinking and the composition of hydrogels and their swelling capacity (Q) and swelling response in different environments. The modulations of hydrogels (swelling/deswelling behavior) in response to changes in some parameters (temperature, electric field, pH, ionic media, etc.) are characteristic to hydrogels sensitive to different external stimuli (stimuli-responsive hydrogels) and are due to changes in polymer–polymer and water–polymer interactions.

*Cellulose-based hydrogels* have outstanding water retention capacities due to the presence of numerous -OH groups in cellulose’s structure. Q can be controlled either by chemical functionalization of cellulose or by choosing a certain synthesis method or a specific type of crosslinking agent [126,127,128]. Thus, the swelling behavior of hydrogels is influenced by the presence of polar and hydrophilic groups in the polymer chains (–OH, –COOH, –CONH_2_, –SO_3_H) and by the degree of crosslinking. Another way to control the Q of hydrogels is to introduce CNCs into their structure, which in addition to network reinforcement, can also modulate the swelling behavior [129]. Q of hydrogels can be designed to respond to certain external stimuli, such as pH, temperature, electric fields, light, etc. [130,131]. Cellulose-based hydrogels can contain a large amount of water or biological fluid, which simulates biological tissue in the healing process. Moreover, Q is a very important property for practical applications of cellulose-based hydrogels, for example, as wound dressings, where a high-water absorption capacity is required, being necessary to fulfill a hemostatic role. Regarding the field of TE, swelling achieves the diffusion of nutrients and other molecules, thus helping cell migration through the hydrogels [69].

*Hemicellulose-based hydrogels* exhibit a lower Q than cellulose-based hydrogels due to the structural differences between these two polysaccharides. However, Q can be improved by changing their network’s structure and crosslinking density, as well as by the incorporation of different functional materials (polymers or nanoparticles) [132]. Usually, weakly crosslinked networks tend to exhibit higher swelling capacities, but they do not show structural stability over time. Recent developments have focused on improving both water absorption and long-term retention. An example is the integration of natural fillers (cellulose nanofibers), which can enhance the hydrophilic nature of the matrix while improving mechanical strength [125]. Other methods are related to surface modifications or blending with polymers (PVA, PAM, chitosan, alginate) in order to prepare composite hydrogels with more balanced swelling behavior [118]. The improvement in water absorption properties of hemicellulose-based hydrogels has an important impact on applications such as those in the biomedical field, providing a promising platform for drug delivery and wound dressing.

### 4.2. Mechanical Properties

The mechanical strength of hydrogels is an important factor in establishing their suitability for applications when structural integrity is crucial. Although biopolymer-based hydrogels exhibit biocompatibility and biodegradability and are inherently sustainable, they cannot achieve the superior performances of synthetic polymer-based hydrogels in terms of durability and mechanical properties. Therefore, an option to achieve a balance between these properties is the use of composite hydrogels, a mixing of natural and synthetic polymers with tunable properties characteristic of both polymers [133,134]. Another promising solution for improving mechanical properties is the incorporation of nanoparticles, such as silica, carbon nanotubes or NCs, into the hydrogel network. The use of different crosslinking strategies demonstrates that these challenges can be successfully overcome. However, all these modifications can complicate the hydrogels’ synthesis process and may introduce additional costs.

While native cellulose is known for its strong tensile properties, *cellulose-based hydrogels* are typically more brittle and exhibit poorer mechanical strength [135]. A simple method to modulate the mechanical properties of hydrogels involves either adjusting the polymer concentration or the crosslinking density, or both. Choosing a crosslinking method (physically or chemically) or the incorporation of reinforcing agents (such as, CNCs, CNFs, CNTs or inorganic nanoparticles) can significantly improve the mechanical properties of hydrogels [136,137]. Regarding the physical crosslinking of hydrogels, this in most cases offers inferior mechanical properties and is susceptible to rapid degradation. If chemical crosslinking is used, they result in superior mechanical strength, which makes them suitable for a wide range of applications [138,139]. Also, the 3D structures of hydrogels can be achieved by dynamic crosslinking (covalent bonds that are not as stable, e.g., Schiff base reactions or thioester bonds), which, if not offering mechanical properties as strong as in the previous cases, still offers good viscoelastic properties, making them mechanically similar to the native ECM and closer to the natural flexibility of body tissues [140,141]. Another option to overcome the inherent limitations of natural polymers is to make interpenetrating polymer network (IPN) hydrogels.

When preparing cellulose-based hydrogels for applications such as controlled drug release, it is necessary to achieve an optimal balance between structural integrity, mechanical flexibility and swelling capacity. For their use in wound care applications, they must be designed to provide superior mechanical properties (resistance to various stresses), but at the same time be sufficiently soft and flexible [142,143]. Another critical network parameter that has a significant influence on the mechanical properties of the hydrogels is the porosity of the network. An appropriate porous structure of the hydrogels facilitates cell adhesion, supports cell migration and differentiation and promotes tissue growth. Hydrogels with good mechanical properties can be used in special cases of tissue repair and regeneration, such as artificial cartilage and skeletal muscle tissues, because these can contribute to structural reorganization and restore initial function [126,144,145,146].

*Hemicellulose-based hydrogels* are generally less mechanically strong than cellulose-based hydrogels. Various strategies have been developed to enhance their mechanical performance, which include chemical modification of the hemicellulose or the tailoring of their 3D networks to meet the proposed requirements [147,148,149,150,151]. The mechanical behavior of these hydrogels is significantly influenced by the crosslinking degree and the choice of crosslinking agents. By tuning these parameters, it is possible to fabricate hydrogels ranging from soft, flexible matrices suitable for biomedical applications to more rigid constructs intended for industrial use. There are different methods to improve the structural integrity and mechanical strength of hemicellulose-based hydrogels [121,124,125]: (i) double crosslinking—the use of mixed crosslinked networks (physically and chemically) leads to interpenetrating polymer networks; (ii) the incorporation of nanofillers can effectively fill void spaces within the hydrogel matrix, thereby increasing its load-bearing capacity and modulus; (iii) salt-induced aggregation—electrolyte-mediated polymer condensation by the Hofmeister effect promotes the formation of denser and more rigid polymer networks; (iv) the use of freeze–thaw cycles—repeated freezing and thawing induces phase separation and hydrogen bond formation, resulting in compact, layered structures; or (v) macromolecular chain extension—grafting hemicellulose with monomers (e.g., acrylic acid, acrylamide) increases the molecular weight and crosslinking density of the hydrogels. These methods, which include network engineering, confer tunable properties to hemicellulose-based hydrogels, which recommends them for specific applications, such as soft actuators, tissue scaffolds or load-bearing biodegradable materials. Even though their mechanical properties can be improved through crosslinking, these hydrogels still struggle to match the strength and stability of cellulose-based variants.

### 4.3. Biocompatibility and Biodegradability

Biodegradability is a crucial feature of the hydrogels, especially in applications where accumulation of synthetic materials can be problematic. Biocompatible hydrogels are of major importance due to the fact that they do not have a rejection response by the body and, in addition, play a positive role in promoting cell adhesion, differentiation and growth.

*Cellulose-based hydrogels* are highly biocompatible (showing both histocompatibility and blood compatibility) due to the natural origin of cellulose. These are non-toxic and generally well accepted by the human body, which makes them ideal candidates for medical applications [126,152]. 

Regarding the biodegradation of cellulose-based hydrogels, it is known that in nature there are various microorganisms, including fungi and bacteria, that can produce cellulases capable of hydrolyzing cellulose. Cellulases can be classified into three distinct classes [153,154,155]: (i) exoglucanase (exo-1,4-β-D-glucanases, CBH), which degrades cellulose by removing cellobiose units from the non-reducing end of the chain, being more active in amorphous or partially degraded substrates; (ii) endoglucanase (endo-1,4-β-D-glucanases, EG), which randomly attacks regions of low crystallinity in cellulose, creating free chain ends and causing rapid chain breakage; and (iii) β-glucosidases (BG), which together with CBH and EG hydrolyze cellulose by cleaving cellobiose and removing glucose from the non-reducing end of oligosaccharides. The enzymatic degradation of cellulose is achieved by the joint action of CBH with EG and BG. The degradation of cellulose or cellulose-based hydrogels in the human body is not possible due to the lack of specific enzymes, especially BG, involved in the cellulose degradation necessary for breaking the β-acetyl linkages [156]. However, cellulose hydrogels can be degraded in the human body through a non-enzymatic process carried out in several stages: (i) initially, the degradation is carried out mechanically in the mouth, then (ii) the hydrogels are chemically degraded in the stomach, (iii) they are grounded and mixed in the small intestine, which is carried out by intestinal peristalsis, and finally, (iv) they are eliminated from the body.

*Hemicellulose-based hydrogels* are increasingly explored for biomedical and environmental applications due to their natural origin [157,158,159]. Recent studies confirm that these hydrogels have excellent biocompatibility and can provide a moist, non-toxic environment that facilitates cell adhesion and proliferation, thus supporting their potential use in TE [122].

Biodegradability is another key advantage of hemicellulose. The degradation takes place in the presence of hemicellulases, represented by xylanases, β-mannanases, arabinofuranosidases and β-xylosidases. The hemicellulose-based hydrogels are susceptible to enzymatic and hydrolytic degradation, breaking down into non-toxic by-products such as monosaccharides and organic acids. The degradation rate can be controlled by modifying the crosslinking density or incorporating other biodegradable natural polymers. This tunability allows their use in applications where gradual material breakdown is desired, e.g., bioresorbable implants. Thus, hemicellulose-based hydrogels offer a promising combination of biological safety and environmental sustainability. Their inherent biocompatibility ensures minimal cytotoxicity and supports tissue integration, while their biodegradability allows for controlled degradation without harmful residues [160]. These characteristics make them strong candidates for future developments in both medical and ecological materials.

### 4.4. Property Comparative Analysis and Challenges

Hydrogels derived from lignocellulosic biomass—cellulose and hemicellulose—have attracted significant attention due to their unique structural characteristics and physicochemical properties. However, each of these types of hydrogels presents advantages and limitations, the knowledge and understanding of which are essential for selecting the appropriate material, method and parameters for using hydrogels in specific applications.

One of the significant challenges of these biopolymers is their *structural compositions and morphologies*. The variability of biopolymer composition represents a significant challenge for standardizing the properties of hydrogels, depending on the source of extraction (woody or non-woody biomass) or on other important aspects (species, tissue type or maturity of the plant cell wall). Efforts to develop standardized processes for the extraction and modification of these polymers are ongoing, but there is still no generally accepted method for achieving consistent hydrogel performance. Although significant progress has been made in improving the *mechanical strength* of hydrogels based on natural polymers, they have not yet reached the performance of hydrogels based on synthetic polymers. Moreover, among these two polysaccharides, cellulose-based hydrogels are the most efficient in terms of mechanical strength. In this regard, future research should focus on strengthening these hydrogels and finding innovative methods to overcome this obstacle so that hydrogels with improved performance (e.g., hybrid hydrogels, smart hydrogels, etc.) can have a significant impact in multiple industries.

Regarding the *cost-effectiveness* of these hydrogels, it is known that the extraction methods (conventional and non-conventional), as well as the crosslinking methods of these biopolymers, are expensive due to their high resource and energy consumption, limiting the large-scale production of the hydrogels. In developing extraction methods, it is necessary to establish a balance between efficiency, environmental friendliness and material quality, which represents a serious challenge that must be taken into account. Although cellulose benefits from relatively well-established isolation methods, the cost of chemical modification, crosslinking and functionalization still represents a major issue. Research on more sustainable and cost-effective extraction, as well as on efficient crosslinking methods, is crucial for the future commercialization of these hydrogels. Hemicellulose-based hydrogels are similarly restricted by the cost and complexity of the extraction methods of hemicelluloses, as well as by their chemical crosslinking methods.

As presented above, biopolymer-based hydrogels have several advantages and disadvantages, which must be taken into account when we intend to use them for a certain medical application (Table 4).

Thus, hydrogels for controlled drug release applications must have a high swelling capacity, which allows for efficient encapsulation of the drugs, in order to further achieve a controlled release of the drugs (with the possibility of either increasing the drug release time or controlling the amount of drug released in a certain time). For wound healing applications, it is necessary to have the ability to maintain a moist environment that promotes rapid healing and scarring and to form a protective barrier against bacterial infection, while absorbing exudates to reduce inflammation. A particularly important aspect in the case of TE applications is the presence of a distinctive 3D network structure of the hydrogels, with well-defined and interconnected pores. Furthermore, the resulting scaffolds must provide mechanical support to promote cell adhesion, proliferation and differentiation and, in the end, implant integration or tissue regeneration.

## 5. Biomedical Applications of Hydrogels Based on Renewable Biopolymers

In the biomedical field, the unique characteristics of cellulose- and hemicellulose-based hydrogels make them valuable materials that can provide versatile solutions to complex challenges encountered. Characteristics such as consistency similar to biological tissues and biocompatibility with living tissues are some of their distinctive properties.

Cellulose-based hydrogels can be used for a wide range of applications due to their characteristics such as their superabsorbent capacity, biodegradability and biocompatibility, robust mechanical strength and cost-effectiveness. Cellulose hydrogels offer the possibility of effectively targeting drug delivery to specific sites in the body. Moreover, their biocompatibility and ability to incorporate antimicrobial agents make them ideal as wound dressings, for treating infections and for promoting wound healing. They also provide a substrate with the necessary characteristics for cell migration and proliferation (porosity and pore interconnectivity), which is particularly important in TE applications [48].

Hemicellulose-based hydrogels represent a class of promising materials that find a wide range of applications in various fields. Hemicelluloses commonly used in these applications are xylan, xyloglucan, konjac glucomannan and galactomannan. Their inherent biocompatibility, biodegradability and ability to form water-absorbent structures recommend them as valuable alternatives to synthetic polymers. Although there are challenges regarding mechanical strength and variability in hemicellulose sources, ongoing research is advancing the development of more robust and functional hemicellulose-based hydrogels. Hemicellulose-based hydrogels exhibit exceptional adsorption capacities due to a high density of OH groups and their ability to form interconnected porous networks, which make them extremely effective for the encapsulation and controlled release of drugs [161]. Furthermore, due to their antimicrobial properties, these hydrogels can form a protective barrier against bacterial infections while helping to reduce inflammation, properties that recommend them for wound healing and tissue engineering applications.

### 5.1. Drug Delivery

Cellulose-based hydrogels can serve as matrices for drug delivery applications due to their ability to absorb large amounts of water and undergo reversible swelling, ensuring a constant therapeutic effect. By tailoring their structure and, implicitly, their specific swelling behavior, the controlled drug release process can be influenced and adapted to specific therapeutic needs [131]. For example, these hydrogels can respond to various environmental stimuli, such as light, temperature, pH and chemical interactions, as well as electric and magnetic fields. These properties, along with their conductivity, elasticity, softness and biocompatibility, are crucial for drug delivery systems, allowing the targeted release of drugs in specific environments, such as acidic tumors or inflamed tissues [162].

In recent years, hemicellulose-based hydrogels have received increased attention for their use in the field of controlled drug release. In order to obtain pH-sensitive hemicellulose-based hydrogels, it is necessary to incorporate specific functional groups (carboxyl and amino groups) capable of protonation or deprotonation in response to pH variations. Specifically, a variation in ambient pH leads to variation in electrostatic repulsion and H-bonding within the hydrogels, thereby affecting the swelling ratio [163].

#### 5.1.1. Cellulose-Based Hydrogels

**Figure 10:**
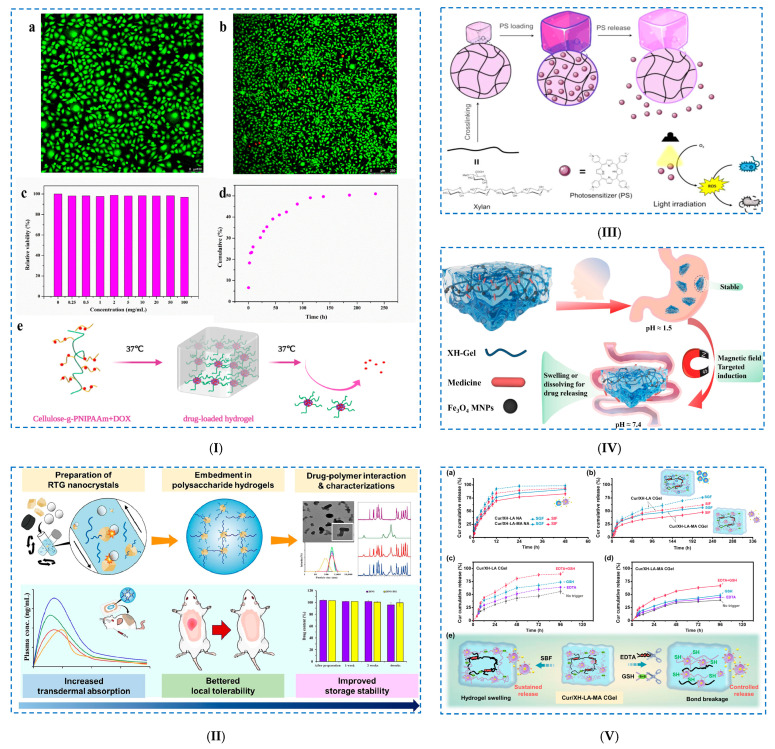
Cellulose- and hemicellulose-based hydrogels as drug delivery systems: (**I**) (**a**) Fluorescence image of L929 cells with 100 mg/mL PNIPAM-g-Cell injectable hydrogel. (**b**) Fluorescence image of L929 cells with 5 mg/mL PNIPAM-g-Cell hydrogel. (**c**) Viability histogram of L929 cells after co-cultivation with PNIPAM-g-Cell hydrogel leaching liquor. (**d**) Release curves of DOX in the PNIPAM-g-Cell hydrogel. (**e**) Schematic diagram of loading and release mechanism of the PNIPAM-g-Cell hydrogel. Reproduced with permission from [109]. (**II**) Schematic illustration of the RTG NS-HG system for transdermal RTG delivery. Reproduced with permission from [169]. (**III**) Schematic illustration of PS-loaded hydrogel synthesis and photocytotoxicity against Gram-negative and Gram-positive strains. Reproduced with permission from [114]. (**IV**) Drug control release mechanism of biobased pH/magnetic dual-responsive Fe_3_O_4_@XH-Gel nanocomposite hydrogel in human tissues. Reproduced with permission from [123]. (**V**) In vitro drug release from Cur-loaded composite hydrogels. Cur cumulative release profiles of Cur/XH-LA nanoaggregates (Cur/XH-LA NA) and Cur/XH-LA-MA nanoaggregates (Cur/XH-LA-MA NA) (**a**). Cur cumulative release profiles from Cur/XH-LA CGel and Cur/XH-LA-MA CGel (**b**). EDTA and/or GSH stimuli-responsive release profiles of aggregates from Cur/XH-LA CGel (**c**) and XH-LA-MA CGel (**d**). Schematic illustration for the Cur release from Cur-loaded XH-LA-MA CGel (**e**). Reproduced with permission from [122].

One of the examples of cellulose-based hydrogels that have found applications as drug delivery systems is one made from a cellulose graft copolymer (PNIPAM-g-Cell) [109]. Its drug release behavior was tested by incorporating doxorubicin (DOX) as a drug, when it was shown to have the ability to release the drug in a long-term and continuous manner for 10 days. Furthermore, the hydrogels also showed good injectability, which confirms that the PNIPAM-g-Cell hydrogel can be used in injectable drug delivery systems (Figure 10I).

Its drug release behavior was tested by incorporating doxorubicin (DOX) as a drug, and it was shown to have the ability to release the drug in a long-term and continuous manner for 10 days. Moreover, the hydrogels also showed good injectability, which confirms that the PNIPAM-g-Cell hydrogel can be used as an injectable drug delivery system. Regarding the cytocompatibility of the hydrogels, it was evaluated against L929 cells. As can be seen in Figure 10I(a,b), the cells showed high activity for 24 h for different concentrations of injectable hydrogel such as 5 mg/mL and 100 mg/mL PNIPAM-g-Cell. These results demonstrate that the injectable hydrogel shows excellent biocompatibility, highlighting its potential for application in drug delivery systems.

Ahmadpour et al. [164] prepared a multifunctional hydrogel for in vitro hyperthermia applications. Hyperthermia treatment is a non-invasive process included in radiotherapy that increases the temperature of the tumor (between 41 and 50 °C) in order to target tumor cells without affecting normal cells. Thus, the local increase in temperature can make cancer cells more susceptible to the effects of radiation and certain anticancer drugs. The obtained hydrogels based on pectin and cellulose (Pec-Cel) contain Fe_3_O_4_ magnetic nanoparticles (MNPs). These new nanobiocomposites exhibit a heterogeneous structure and a saturation magnetization value of 48.80 emu g^−1^, demonstrating that they may be good candidates for in vitro hyperthermia processes. It was observed that the highest specific absorption rate (SAR) was obtained for the sample with the lowest concentration of magnetic nanobiocomposites, explained by the presence of dipole–dipole interactions between MNPs, which could disrupt their heat generation. Moreover, by increasing the sample concentration (from 0.5 mg/mL to 10.0 mg/mL), a significant reduction in SAR was observed (from 126.0 W g^−1^ to approximately 5.0 W/g). The samples were also exposed to an alternating magnetic field (AMF) at different frequencies to evaluate their capacity for an in vitro hyperthermia process. A less pronounced influence was observed compared to the sample concentration on SAR and the effect of the AMF frequency differed depending on the sample concentration.

Bicomponent hydrogels based on cellulose (C) and dextran (D), which exhibit anti-inflammatory properties, were obtained by incorporation of polyphenols (PFs) extracted from Chambourcin grape seeds [165]. It was observed that the introduction of D into the matrices had a positive impact on the morphology of the hydrogel by increasing the uniformity and interconnectivity of the pores, as well as the swelling capacity of the hydrogels. The CD25/75 hydrogel (25% C and 75% D), which has the highest swelling degree (4520%), exhibits a significantly higher PF encapsulation capacity compared to other cellulose-dextran hydrogels. It was observed that the PF release process depends on the composition of the hydrogels and is carried out over a long period of time (3 days), indicating a controlled and prolonged release of the anti-inflammatory bioactive compound. Biocompatibility tests (fibroblasts and endothelial cells) showed a high viability for all formulations (>80%). Furthermore, anti-inflammatory properties of PF-encapsulated CD hydrogels were proven in the presence of human cells stimulated with lipopolysaccharide (LPS, a pro-inflammatory model). The results showed that there was a nearly 100% reduction in inflammation at a concentration of 50 g/mL of PF.

Tang et al. [166] successfully synthesized an injectable and self-healing hydrogel made of biocompatible poly(N-vinylpyrrolidone)/carboxymethyl cellulose (PVP/CMC). This hydrogel was designed for the controlled release of 4-aminosalicylic acid (4-ASA), a drug used in the treatment of tuberculosis and inflammatory bowel diseases. In the preparation of the hydrogel, MBA was used as a crosslinking agent and potassium persulfate as an initiator. The resulting hydrogels demonstrated a good swelling ratio, which increased with the increase in the amount of CMC and reached a maximum of 2850% (PVP:CMC = 1.0:1.5). It was observed that the pH value is an important factor in the drug encapsulation process. Thus, the drug loading increased from 2.5 (pH 2.0) to 3.5 (pH 7.4), explained by the reduced interference of H-bonds in a less acidic environment and by the protonation of the carboxyl groups of CMC at low pH, as well as the formation of hydrophobic regions, which caused the hydrogel network to contract and prevent drug diffusion. Overall, this study demonstrated that CMC concentration and pH significantly influence the loading and drug release behavior of PVP/CMC hydrogels, making them promising for controlled drug delivery applications.

A colon-targeting oral drug delivery system was prepared by incorporating N-acetylglucosamine (GlcNAc) into a methylcellulose (MC) hydrogel, which was then encapsulated in a 3D-printed shell composed of a mixture of methyl acrylate–methyl methacrylate–methacrylic acid terpolymer (EURFS100) and polylactic acid (PL) [167]. The influence of the polymer mixing ratio was evaluated, and it was observed that a lower amount of PLA implied a significantly better controlled drug release and more reproducible printing. In addition, it was demonstrated that a higher concentration of MC hydrogel resulted in better control over drug release and gelation behavior. Following this study, it was concluded that tablets with 80/20 wt% Eudragit^®^ FS100/PLA and the drug-loaded hydrogel with 30 mg/mL GlcNAc and 3% *w*/*v* MC showed the best results, such as the best printability, processability and drug release kinetics.

Cutaneous and transdermal drug delivery represent effective alternatives to traditional drug delivery routes. Cutaneous delivery targets the deeper layers of the skin, where the drug only penetrates the outer layer of the skin (stratum corneum), while transdermal delivery facilitates drug transport to the dermis before entering the systemic circulation. These methods offer several advantages, including sustained release at a constant rate, suitability for self-administration and avoidance of first-pass hepatic metabolism and gastrointestinal incompatibilities [168].

Park et al. [169] prepared a skin-friendly nanocrystalline suspension (NS)-loaded hydrogel (NS-HG) for the transdermal delivery of rotigotine (RTG), a non-ergot dopamine agonist used for managing early-stage Parkinson’s disease (Figure 10II). RTG-loaded NS-HGs were fabricated using a laboratory-scale bead milling technique and incorporated into the hydrogel matrix, employing sodium carboxylmethyl cellulose (Na.CMC) as a nano-suspending agent. It was observed that the Na.CMC was uniformly dispersed and that RTG was immobilized in the hydrogel matrix by electrostatic interactions. The novel NS-HG exhibited a transdermal absorption rate comparable to that of the microemulsion. Thus, the transdermal absorption in rats increased proportionally with the drug loading. Furthermore, the NS-HG system demonstrated a significant reduction in skin irritation after repeated applications without causing edema or changes in epidermal thickness due to hyperplasia.

#### 5.1.2. Hemicellulose-Based Hydrogels

Composite hydrogels based on hemicellulose from bagasse were prepared by using PDA microspheres as reinforcing agents [170]. PDA microspheres filled the voids in the 3D network of the hydrogels, contributing to increasing their density and improving their mechanical properties (with a maximum compressive strength of 0.30 MPa). Due to the fact that PDA is insoluble in water, the water retention capacity of the composite hydrogels decreased from 94.42% to 51.49%. However, the capacity of the composite hydrogel to adsorb the drug (methylene blue) was found to be higher (0.41 mg/g) than the traditional one (0.35 mg/g). Regarding the drug release, it was 62.82% for the composite hydrogel, compared to 47.77% for the traditional hydrogel. Furthermore, the pH dependence of the drug release was demonstrated, which could be adjusted to reduce the side effects of drugs in the stomach and to be released directly into the intestine.

Hydrogels that respond to specific stimuli (light, temperature or redox potential) can be used to deliver drugs to certain tissues affected by disease without damaging unaffected tissues [171].

A light-sensitive hydrogel based on beech xylan was developed by incorporating the hydrophilic photosensitizer 5,10,15,20-tetrakis(1-methylpyrid inium-4-yl)-porphyrin tetraiodide (TMPyP) [114] (Figure 10III). This hydrogel showed an interconnected porous structure, a high swelling ratio (<62), good mechanical properties and a prolonged release of PS (<24 h), with a drug cumulative amount of 100%. Moreover, the TMPyP-loaded hydrogel showed a photocytotoxic effect against *Pseudomonas aeruginosa*, *Escherichia coli*, *Staphylococcus aureus* and *Bacillus cereus*. Wang et al. [125] prepared an injectable hydrogel by light-induced thiol-ene crosslinking. The hydrogels obtained from between methacrylate-modified O-acetyl-galactoglucomannan (GGMMA) and thiolated cellulose nanocrystal (CNCs-SH) demonstrated strong mechanical properties and fast enzymatic degradation. The incorporation of bioactive glass nanoparticles (BaGNPs) into the hydrogels led to the attainment of therapeutic ion delivery systems (Si and Ca ions/species) that could be released into the human body.

A redox-sensitive hydrogel was constructed via a UV-triggered thiol–disulfide exchange reaction, a reversible dynamic nanocomposite hydrogel composed of thiolated galactoglucomannan (GGMSH) and TEMPO-oxidized cellulose nanofibrils (T-CNFs) [172]. The resulting GGMSH/T-CNF hydrogel demonstrated tunable mechanical strength, good cytocompatibility, self-healing properties and a rapid BSA release profile. These hydrogels could be used as a delivery vector for the controlled release of therapeutics.

A pH/magnetic dual-responsive hydrogel was prepared through the one-step in situ polymerization of xylan hemicellulose (XH), acrylic acid, PEGDA and Fe_3_O_4_ magnetic nanoparticles [123] (Figure 10IV). The Fe_3_O_4_@XH-Gel nanocomposite hydrogel recorded a maximum Seq of 207.8 (AA:XH = 12:1) and a pH-depending swelling ratio that varied from 5.0 (pH = 2) to 48.9 (pH = 8). An excellent capacity for constant and continuous release of drugs (acetylsalicylic acid and theophylline) was also demonstrated, even under fluctuating pH conditions. Magnetic hydrogels offer faster responses and more precise control than hydrogels sensitive to stimuli such as pH or light, giving them a crucial role in controlled drug delivery [173].

Konjac glucomannan (KGM)-based hydrogels, although they have significant potential for use in targeted drug delivery systems due to properties such as high viscosity, solubility, non-toxicity and gelation, unfortunately present certain challenges in terms of water absorption behavior and controlled drug release characteristics [174]. In an attempt to overcome these shortcomings and improve the properties of KGM-based hydrogels, Wu et al. [175] used oxidized hyaluronic acid (OHA) to obtain composite hydrogels. After evaluating the hydrogels, it was observed that the KO-2 hydrogel, which contained a concentration of 0.3% OHA, had the most stable network structure and the best mechanical properties. Epigallocatechin gallate (EGCG) was also incorporated into the KGM/OHA hydrogels to study the release kinetics of the model drug. It was demonstrated that the highest entrapment efficiency was recorded by the KO-2 hydrogel (22.40 ± 1.24%), and the amount of EGCG released after 10 h was 57.62 ± 4.11%. Thus, it was observed that in the presence of OHA, a significant improvement could be obtained in both the EGCG encapsulation efficiency and the release properties of the hydrogels. All these results confirm that the properties of the composite hydrogel can be controlled by the OHA content and that the obtained hydrogels are capable of controlled and long-term release of drugs, which can be used for biomedical applications.

Shen et al. [122] encapsulated the hydrophobic drug (curcumin) in covalently crosslinked hemicellulose laurate methacrylate (XH-LA-MA) nanoaggregates (Figure 10V). In the next step, the drug-loaded nanoaggregates were incorporated in a double-network hydrogel based on alginate/Ca^2+^ and P(AA-co-DMAEMA). This composite hydrogel demonstrated stimuli responsiveness to EDTA and/or glutathione (GSH), and was able to disrupt the network of the hydrogel through chemical interactions, thus achieving the controlled release of curcumin. All the composite hydrogels were biocompatible (NIH/3T3 cells).

A summary made for the last 5 years regarding the main characteristics, as well as the biological properties, of cellulose- and hemicellulose-based hydrogels that have applications in the field of drug delivery systems is presented in Table 5.

### 5.2. Wound Healing

Wound dressings are known for their excellent properties, such as their superior absorbency, easily moldable nature, flexibility and elasticity, adhesiveness, biocompatibility and extreme versatility, properties that allow their use in treating a wide range of wounds, such as abrasions, lacerations, surgical incisions, burns, pressure ulcers and chronic wounds [176,177,178]. An important fact is that hydrogel dressings must maintain their structural integrity throughout the healing process. Self-healing hydrogels have emerged as an innovative approach following the increased demand for materials that mimic natural systems and independently repair their structural and functional damage. Dynamic and reversible chemical interactions are used to create these crosslinked hydrogel networks. Self-healing hydrogels can be classified into physical and chemical, depending on their healing mechanisms [179].

Cellulose-based hydrogels can provide a moist environment that promotes wound healing and prevents infection. They also have the capacity to absorb exudates from wounds and facilitate the controlled release of antimicrobial agents or growth factors. However, as clinical demands for improved performance in wound healing have evolved, hydrogel dressings with enhanced single or multiple biological activities have emerged. In addition to the possibility of in situ production and rapid hemostasis, hydrogels serve as essential physical barriers, preventing external bacterial infections [180,181,182,183].

Hemicellulose-based hydrogels have gained prominence as wound dressings due to their anti-inflammatory effects, immune functionality and antioxidant properties, as well as their ability to maintain a moist environment that promotes wound healing and scarring [184,185].

#### 5.2.1. Cellulose-Based Hydrogels

Guamba et al. [186] extracted and characterized cellulose from plants originating from Ecuador: pear mesocarp (F1), pear epicarp (F4), tomato (F12) and pitahaya (F53). The celluloses thus obtained were compared with a commercial cellulose. All these celluloses were used to prepare hydrogels (was not possible with F1), which were tested in vitro for antimicrobial properties and evaluated for applications in wound dressings (pig skin model). It was shown that hydrogels synthesized from F53 had the greatest potential in inhibiting bacterial proliferation due to their antifouling properties, which prevented the adhesion and growth of bacteria. Considering its resistance to bacterial growth, F53 proved to be the most promising hydrogel for wound dressing preparation, outperforming both commercial cellulose-based hydrogels and standard wound dressings. However, F53 exhibited reduced bacterial growth in the ex vivo pig skin model. This discrepancy was likely due to the variation in bacterial strains present in the pig skin samples compared to the single-strain inoculum used in the in vitro evaluations.

**Figure 11:**
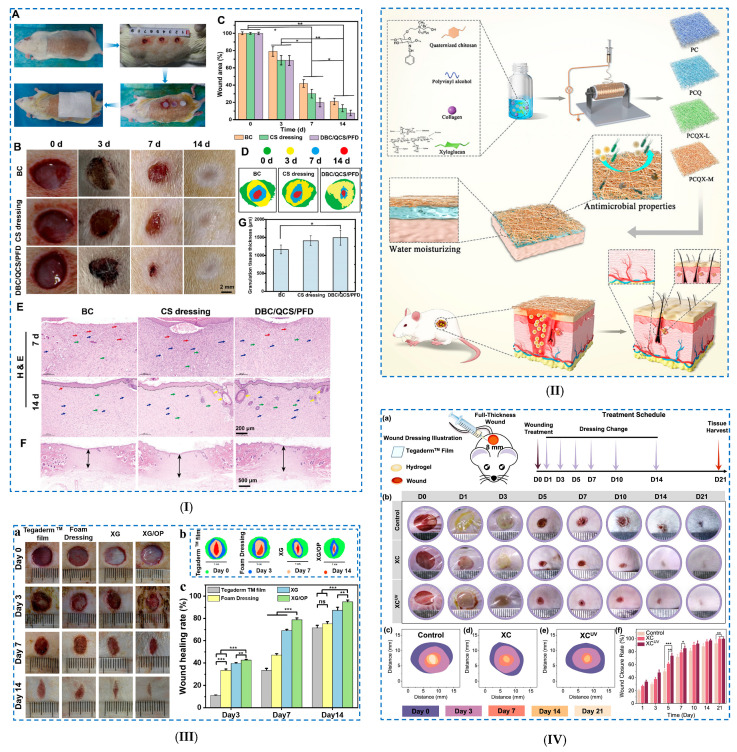
Cellulose- and hemicellulose-based hydrogels in wound heling: (**I**) (**A**) Rat wound modeling. (**B**) Photographs of the wound healing process. (**C**) Statistical analysis of wound area. (**D**) Schematic picture of wound healing in rat model. (**E**) Images of H&*E*-stained wound parts (dark blue arrow for blood vessel, green arrow for fibroblast, red arrow for neutrophil and yellow arrow for hair follicle). (**F**) Images of granulation tissue thickness. (**G**) Histogram of granulation tissue thickness, treated with BC, CS dressing and DBC/QCS/PFD hydrogels. Reproduced with permission from [188]. (**II**) Schematic illustration of electrospun nanofibrous dressings for infected wound healing. Reproduced with permission from [192]. (**III**) (**a**) Photographs of rats with full-thickness wounds treated with Tegaderm™ film, foam dressing, XG hydrogel and XG/OP hydrogel. (**b**) Schematic of wound bed closure. (**c**) Wound healing rate. Reproduced with permission from [194]. (**IV**) (**a**) Schematic illustrating the wound and treatment schedule in mice. (**b**) Representative images of cutaneous wounds treated with XC, XCUV and saline solution. The schematic diagram of the dynamic wound healing process for (**c**) control, (**d**) XC and (**e**) XCUV hydrogel groups. (**f**) Wound closure rate from day 1 to day 21 (n ≥ 3). Data are reported as means ± SD (* *p* < 0.05, ** *p* < 0.01, *** *p* < 0.001, n ≥ 3). Reproduced with permission from [195].

Yi et al. [187] synthesized a hydrogel dressing for wound regeneration with multiple functions, such as adhesive, antibacterial, hemostatic and self-healing. This hydrogel was made of BC, PVA, polydopamine (PDA) and borax (PB) and loaded with doxycycline hydrochloride (Doxy). To improve the mechanical strength of the hydrogels prepared from dynamically crosslinked PVA by ester linkage with PB, a reinforcing material made of PDA@BC (dopamine-induced self-polymerization) was introduced. The resulting hydrogels, PB-PDA@BC loaded with Doxy, were proven to exhibit exceptional antibacterial properties, along with favorable cytocompatibility and blood compatibility. In addition, the PB-PDA@BC/Doxy hydrogel showed significant wound healing, reducing the wound size by 20.1% on day 3 (*p* < 0.05), and on day 14, almost complete healing was recorded (97.1% wound closure). Regarding the evaluation of the wound healing process by histological analysis, it was observed that the PB-PDA@BC/Doxy hydrogel presented a lower number of inflammatory cells compared to the control group, an explanation being the presence of Doxy and its antibacterial effect. Moreover, on day 14, the presence of hair follicles and blood vessels was recorded for the PB-PDA@BC/Doxy hydrogel, which proves its ability to promote skin regeneration and accelerated tissue repair. Deng et al. [188] prepared a hydrogel (DBC/QCS) from dialdehyde bacterial cellulose (DBC) and quaternized chitosan (QCS) that demonstrated a superior swelling capacity and structural stability, tested by repeated cycles of dehydration and rehydration (Figure 11I). Thus, the hydrogel could swell upon rehydration to values over 1000% without affecting its mechanical integrity. Antimicrobial tests against *E. coli* and *S. aureus* showed strong antibacterial efficacy, being able to eliminate over 80% of bacteria, a fact explained by the presence of the positively charged QCS. An increase in fibroblasts (biocompatibility tests) and wound healing (in vivo evaluations), but also an increased collagen deposition and a reduction in scars (in the presence of pirfenidone), were recorded.

All this information confirms that cellulose-based hydrogels have experienced a rapid evolution from classic wound dressings to dynamic therapeutic platforms, offering distinct advantages for complex environments and each type of wound.

#### 5.2.2. Hemicellulose-Based Hydrogels

An adhesive hydrogel based on xylan, polyacrylic acid (PAA) and tannic acid (TA) was fabricated [189]. It is known that both xylans and TA exhibit unique properties, such as antioxidant and antibacterial properties. The obtained hydrogels demonstrated excellent elasticity (fracture strain of <4600%), toughness (fracture energy of 1954 J/m^2^) and adhesive strength (<25 kPa), but also biocompatibility (NIH-3T3 fibroblast cells) and excellent antioxidant and antibacterial properties.

Composite hydrogels made by crosslinking arabinoxylan (ARX), chitosan (CS) and reduced graphene oxide (rGO) in the presence of tetraethyl orthosilicate (TEOS, as crosslinking agent) were fabricated and evaluated as wound healing dressings [190]. The hydrogels were shown to be pH sensitive, with maximum swelling observed at pH 7.4 (due to the electrostatic repulsion of the alcoholate and carboxylate ionic groups). Furthermore, all composite hydrogels exhibited a high swelling capacity (>80%), demonstrating their suitability for wound dressing applications. The release of the antibacterial drug (silver sulfadiazine) was also shown to be pH-dependent, with maximum release (93.1%) at pH = 7.4 and minimum (58.3%) at pH = 6.4. MC3T3-E1 cells adhered, proliferated and differentiated well on the composite hydrogels, with excellent viability (91%) and appropriate morphologies. Ag and TiO_2_ nanoparticles were loaded onto a xylan/chitosan conjugated matrix to obtain materials that could be used as wound dressings [191]. It was shown that by incorporating nanoparticles, not only the pore size but also the swelling ratio and the adsorption capacity of proteins in the matrix could be modified. Furthermore, it was demonstrated that the materials promoted cell attachment and antibacterial properties of the scaffold and had a strong antimicrobial response against both Gram-negative and Gram-positive bacterial strains. However, some cytotoxicity was observed at high concentrations of Ag nanoparticles.

A multifunctional electrospun nanofiber dressing (PCQX-M) based on xyloglucan, quaternized chitosan, PVA and COL was developed using electrostatic spinning technology [192]. The material was designed to exhibit superior moisturizing properties and promote healing of infected wounds by introducing xyloglucan and quaternized chitosan (Figure 11II). PCQX-M demonstrated good mechanical properties (4.90 ± 0.15 MPa), adequate hemolysis rates (0.94 ± 0.12%) and high biocompatibility (L929 cells), meeting the necessary conditions for wound healing. In addition, PCQX-M demonstrated exceptional antibacterial properties, with an inhibition rate of 96.74% (*E. coli*) and <98% (*S. aureus*) and a potential inhibitory effect on the growth of microorganisms in infected wounds. Furthermore, a significant promotion of cell proliferation and angiogenesis at the injury site, as well as healing of the infected wound (murine model of whole skin injury infected with Staphylococcus aureus), was observed.

Xu et al. [193] developed a composite hydrogel made of xyloglucan (XG—anti-inflammatory and antibacterial properties) and Pluronic F127 diacrylate (F127DA—photocurable properties) for wound healing. In vitro analysis showed that XG significantly promoted cell proliferation and migration (L929 fibroblasts), also inducing an anti-inflammatory effect. Thus, on day 3, 1%XG/F127DA had the highest wound closure rate across the entire culture (98.30 ± 1.83%), followed by 0.5%XG/F127DA (91.82 ± 1.00%). In the case of in vivo experiments, it was observed that XG/F127DA hydrogels promoted re-epithelialization and angiogenesis, allowing the healing of chronic wounds. Thus, for 0.5% XG/F127DA, granulation tissue thicknesses of 42.90 ± 3.22 μm (on day 9) and 67.24 ± 7.33 μm (on day 12) were recorded.

A hydrogel dressing was made from okra polysaccharide (OP) and xyloglucan (XG) based on a dynamic network of borate bonds, which conferred it with injectability and self-healing properties [194]. The XG/OP composite hydrogels exhibited good rapid self-healing properties (the recovery rates: 100%, 99.4% and 98.5% for seven cycles) and the ability for shear thinning, proving to be useful in irregular injured areas. In addition, the XG/OP hydrogels were shown to exhibit antioxidant properties (DPPH of 73.9%), and in vivo experiments demonstrated that they could accelerate wound healing by promoting collagen deposition and angiogenesis. Figure 11III shows the results obtained over 14 days of treatment regarding wound bed closure. It can be seen that for XG/OP, the average healing rate was up to 94.5%, while the Tegaderm™ film group had a wound healing rate of only 71.6%. A new polysaccharide-based hydrogel based on oxidized xyloglucan (OXG) and chitosan amino methacrylate (CSMA) residues was developed to promote complete healing of cutaneous wounds in a mouse model [195]. The structure of the hydrogel consisted of a crosslinked network formed by dynamic covalent crosslinking bonds. The obtained hydrogel was shown to have shear thinning and self-healing properties, which are necessary for in situ gelation in clinical injection procedures. When the methacrylate groups were photoinitiated and polymerized after injection, a second covalent crosslinking network was obtained, a three-dimensional elastically tunable structure for deep wounds. The produced composite hydrogel demonstrated faster wound healing, accelerated angiogenesis, re-epithelialization and hair follicle formation (Figure 11IV). Chen et al. [196] developed an in situ method for preparing BC-based hybrid hydrogels containing dextran (BC-D) and xyloglucan–dextran (BC-XG-D). The obtained hydrogels exhibited good mechanical properties (maximum tensile stress: 1.67 MPa; breaking strain: 52.9%) and were observed to be more stable and softer under high shear deformation. Animal wound healing experiments demonstrated that BC-XG-D hybrid hydrogels were more effective in promoting wound healing and skin maturation.

Dialdehyde carbohydrates (DCs) have been used in various biomedical applications, especially in wound healing, due to their exceptional properties, such as high bioactivity and reactivity, but also designed characteristics [197]. The presence of dialdehyde groups in the carbohydrate structure allows for the crosslinking of macromolecular chains and the formation of versatile 3D architectures for biomedical applications. An example of this is dialdehyde xylan (DAX), which is used to stabilize the dispersion and control the size of silver nanoparticles in hydrogels. DAX was used in the preparation of an antibacterial hydrogel, both as a crosslinking agent for chitosan (CS) and as a stabilizing agent for AgNPs [198]. AgNP size was controlled by reducing the AgNO_3_ solution in a methanolic suspension of the metal–organic framework (MOF) -UiO-66-NH2 and forming a core–shell structure Ag@UiO-66-NH2 that avoided AgNP agglomeration. The obtained hydrogels showed good water absorption (SR < 1100%) and self-healing capacities (efficiency < 88%, after 5 h). Furthermore, the hydrogels were shown to exhibit excellent antibacterial properties, low cytotoxicity and good cell viability (NIH/3T3 cells, survival rate > 90%).

Konjac glucomannan (KGM) exhibits unique properties, such as solubility and gel-forming properties; biological properties, such as antioxidant, anti-inflammatory and antitumor properties; and hypoglycemic and hypolipidemic properties. KGM can induce macrophage polarization (from inflammatory to anti-inflammatory), which leads to the release of anti-inflammatory factors and, consequently, to the acceleration of chronic wound healing [199]. These properties make it an attractive compound for biomedical applications, especially in digestion and metabolic regulation [200]. In addition, KGM is considered an attractive material for wound healing and wound management [201,202,203]. Another example in this regard is the composite hydrogel synthesized from hyaluronic acid (HA) and KGM via the Schiff base reaction [204]. The HAKGM hydrogel demonstrated antibacterial activity (eliminating in 48 h: 75.3 ± 4.9% of *E. coli* and 98.5 ± 4.9% of *S. aureus*) and biofilm activity against the tested microorganisms. Evaluations of the cytotoxicity of HAKGM on NIH/3T3 cells showed that it has excellent biocompatibility (cell viabilities >70%, after 72 h). Furthermore, the hydrogel demonstrated accelerated healing in a full-thickness skin defect model (healing ratio: 98 ± 4.9%, 12 days) and a reduction in the recovery time of burn wounds. A wound dressing (KGM-GA) made of konjac glucomannan (KGM) and gallic acid (GA) was synthesized to accelerate wound healing without the addition of other drugs [205]. This in vitro study demonstrated that KGM-GA could regulate M2 macrophage polarization and reduce reactive oxygen species (ROS) levels, indicating the presence of superior anti-inflammatory properties. KGM-GA also exhibits good biocompatibility (towards Raw 264.7 and L929 cells) and contributes to wound healing by accelerating wound closure, collagen deposition and angiogenesis.

Galactomannan (GM) exhibits hydrophilicity and adhesive properties [206], promotes tissue repair by inhibiting pro-inflammatory cytokines (IL-1β, IL-6) and accelerates healing by increasing TGF-β to stimulate fibroplasia [207]. Souza et al. [208] evaluated the healing activity of a GM gel from *Cassia grandis* seeds, associated or not associated with photobiomodulation therapy (PBMT), in second-degree burns. For this purpose, the authors established four working groups: Control (CG), Gel (GG), Laser/PBMT (LG) and Laser + Gel (GLG). The samples were shown to have excellent biocompatibility (L-929 cells), regardless of the GM concentration. Thus, cell viability was 100 ± 0.67%, for the lowest GM gel concentration (1.5 μg/mL), while for the highest GM gel concentration (50.0 μg/mL), cell viability was 99.42 ± 0.42%. GM also demonstrated significant therapeutic potential for treating burns. After 21 days, all groups demonstrated high recovery, with GLG standing out with the best evolution of epithelial recovery. In combination with Laser/PBMT, GM can synergistically improve tissue regeneration by promoting re-epithelialization and matrix remodeling.

A hydrogel based on galactomannan (from *Cassia grandis* seeds) and κ-carrageenan was prepared by crosslinking with CaCl_2_, with the aim of being used as wound dressings for second-degree burns [209]. To improve their potential for use, the obtained matrices were incorporated with lactoferrin (role in regulating the immune system and antimicrobial and viral activities) and with Cramoll (efficacy in repairing experimental lesions, has an immunomodulatory profile and stimulates cell proliferation). The studied samples were hydrogel (H), hydrogel + lactoferrin (HL), hydrogel + Cramoll (HC) and hydrogel + lactoferrin + Cramoll (HLC). The multifunctional matrix could accelerate the healing of second-degree burn wounds, achieving tissue repair. Histopathological studies indicated that the samples promoted re-epithelialization of wounds, with the contraction of lesions (14 days) being of 20.76% (S), 32.85% (H), 36.05% (HC), 45.49% (HL) and 66.10% (HLC). An increased presence of lymphocyte cells was also observed, indicating rapid healing and prevention of a prolonged inflammatory phase.

Yuyu et al. [210] prepared a hydrogel from aldehyde galactomannans (OGM), HA, AM and [2-(methacryloyloxy)ethyl] dimethyl-(3-sulfopropyl) ammonium hydroxide (MEDA) by radical copolymerization and Schiff base reaction, and it exhibited biocompatibility and self-healing properties. The structure of the hydrogel was achieved by multiple crosslinks (hydrogen bonds, ionic interactions, reversible Schiff base bonds and molecular chain linkage), which gave it excellent mechanical strength and self-repair capacity. The hydrogel exhibited high tensile strength (25 kPa), tensile strain (2200%), toughness (391.59 kJ/m^3^) and Young’s modulus (9.77 kPa). In vivo and in vitro experiments demonstrated that the optimized hydrogel formula exhibited a low inflammatory response, facilitating rapid healing of full-thickness skin wounds (rat models).

A summary made from the last 5 years regarding the main characteristics, as well as the biological properties, of cellulose- and hemicellulose-based hydrogels that have applications in the field wound dressings is presented in Table 6.

### 5.3. Tissue Engineering

Tissue engineering (TE) has become one of the most promising medical solutions for the regeneration of skin, blood vessels, muscles and bones [211]. However, TE requires hydrogels that fulfill specific criteria, balancing physical properties such as degradation rates and mechanical strength with biological performance indicators, like cell adhesion [212]. Biocompatibility is a crucial property, indicating that hydrogels can exist in the body without causing adverse reactions or damage. Any inflammatory reaction to the hydrogel could influence the immune response to the transplanted cells and vice versa [116].

Cellulose and its derivatives exhibit numerous attractive properties that make them suitable for applications such as TE, including biocompatibility, biodegradability, low toxicity or even no toxicity, low cost-effectiveness and excellent mechanical performance [41,213]. Cellulose-based hydrogels are used as scaffolds for TE due to their structural similarity to the natural extracellular matrix. These hydrogels support cell growth, proliferation, differentiation and, implicitly, tissue regeneration [214]. In recent years, nanocellulose hydrogels have been widely used in the field of TE due to their unique 3D porous structure, excellent mechanical properties and good biocompatibility [215,216,217,218].

Hemicellulose-based hydrogels have demonstrated potential for use as bioscaffolds in TE due to their 3D network structure and mechanical support, as well as their possibilities for cellular proliferation and differentiation.

#### 5.3.1. Cellulose-Based Hydrogels

**Figure 12:**
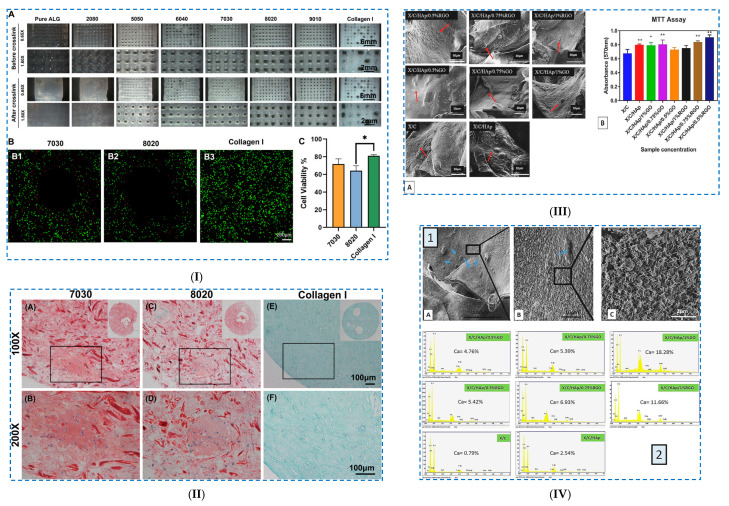
Cellulose- and hemicellulose-based hydrogels in tissue engineering: (**I**) (**A**) Printed mesh structures using different formulations of TCNF/ALG precursors. (**B**) Live/dead images of the (**B1**) 7030, (**B2**) 8020 and (**B3**) COL bioinks. Live cells appear green and dead cells, red. (**C**) Quantitative cell viability of the live/dead assay images. Reproduced with permission from [219]. (**II**) Safranin-O staining (red, stained negatively charged proteoglycans and the TCNF) with Fast Green counterstain (green, stained proteins) and cells stained with hematoxylin of (**A**,**B**) 7030, (**C**,**D**) 8020 and (**E**,**F**) COL I bioinks. Scale bar is 100 μm. Reproduced with permission from [219]. (**III**) (**A**) Cell attachment study and (**B**) MTT assay on different biocomposite scaffolds. Reproduced with permission from [224]. (**IV**) (**1**) SEM image showing self-assembly of deposited minerals on scaffold’s surface, (**2**) EDX spectra revealing percentage calcium deposited on scaffold’s surface when cultured for 72 h. Reproduced with permission from [224].

Lan et al. [219] designed a TEMPO-oxidized CNF (TCNF)- and alginate (ALG)-based hydrogel, incorporating human meniscal fibrochondrocytes (hMFCs), which were harvested after surgical removal of partial meniscectomy (Figure 12I).

They used the initial formulations as potential bioinks, and after identifying the optimal formulation, the hydrogels (prepared by crosslinking with CaCl_2_) were evaluated as a 3D-bioprinted meniscus-like tissue construct. An in vitro evaluation was performed for 6 weeks under hypoxic conditions (3% O_2_) and was compared with collagen I (COL, bovine) as a reference material. The formation of a dense ECM layer on the surface of the cellulose nanofibers was observed, confirmed by histological (Figure 12II) and immunofluorescence results, where it was concluded that TCNF/ALG bioinks loaded with hMFCs support the development of a fibrocartilage-like phenotype of the inner meniscus, while COL bioink supports an outer meniscus phenotype.

Cellulose-based hydrogels are frequently combined with antibacterial components (such as Ag, ZnO and tannic acid), antibiotics (for sterilization) or various polymers to create highly efficient materials to repair and regenerate bone tissue. The introduction of inorganic NPs, such as LAPONITE^®^, hydroxyapatite (HAp) and titanium dioxide, can further enhance cellulose-based hydrogels for bone TE applications [220].

Li et al. [221] made a layered material from a thermosensitive hydrogel (CGHH@NT) prepared from chitosan (CS), glycerol (GLY) and HPMC that was deposited on titanium dioxide (TiO_2_) nanotubes. It should be noted that the hydrogels are in the sol state at a body temperature of 37 °C, while at a body temperature of 40 °C, the hydrogel passes into the gel state. The evaluation of biocompatibility and the effect of osteogenesis was made on osteoblasts (MC3T3-E1) and macrophages (RAW264.7), and cell adhesion and proliferation and the promotion of tissue repair were demonstrated. Furthermore, the activity of alkaline phosphatase (ALP), a protein that acts as an early marker of bone formation during osteoblast differentiation, was studied. It was found that after 7 days of cultivation, no difference in the ALP activity of the samples appeared, but after 14 days, a significantly higher ALP activity was observed in osteoblasts from the CGHH@NT samples.

Tohidi et al. [222] synthesized a composite hydrogel based on collagen and hyaluronic acid, functionalized with oxidized bacterial cellulose grafted with 3-aminopropyl-triethoxysilane (APTES) and gold nanoparticles. It should be noted that the hydrogel formed rapidly at physiological temperature (approx. 2 min) and demonstrated a remarkable self-sealing response (efficiency < 80%) and high cytotoxicity of H9C2 (viability > 90%, after 72 h of incubation). Furthermore, in vitro extracellular field potential tests confirmed the possibility of using the hydrogel as an injectable scaffold for cardiac TE, being able to electrically stimulate human stem cells (47 beats/minute) with a cellular discharge of 5.47 μv. All this information confirms that this hydrogel can be used as a multifunctional platform for cardiac TE.

#### 5.3.2. Hemicellulose-Based Hydrogels

Double network (DN) hydrogels based on xylan, PVA and borax were prepared by physical and chemical crosslinking [223]. The addition of xylan to the composition resulted in the formation of an interconnected 3D network with larger pores, with improved mechanical strength and hydrogel stability. Also, the dynamic PVA–borate network gave the hydrogel ultra-fast self-repair properties, thus eliminating the possibility of breakage after damage. The obtained hydrogels exhibited excellent properties, such as high strength (81 kPa), high toughness (1652.42 kJ/m^3^), good self-recovery (79% recovery) and self-repair properties (85.8% efficiency, for 30 s). A composite scaffold made up of xylan, chitosan, nanohydroxyapatite (HAp) and graphene oxide/reduced graphene oxide (GO/RGO) was prepared for bone TE application [224]. Hydrogels exhibited a morphology with interconnected pores (porosity: 90–93%), similar to a honeycomb structure. The pore size demonstrated that it was beneficial for nutrient exchange and for achieving angiogenesis and osteoinduction in the human body (Figure 12III). Furthermore, it was observed that the addition of GO in the system led to an improvement in the mineralization tendency of apatite, demonstrating an excellent osteogenic capacity (Figure 12IV). The synergistic effect of HAP and GO allowed an increase in cell viability (of MG-63 cells) and osteogenic differentiation.

A thermoreversible hydrogel was made from a xyloglucan derivative (mXG) and an anti-C5a receptor antibody (anti-C5aRab), administered intravenously, for use in the prevention of postoperative peritoneal adhesions [225]. Studies have shown that the obtained hydrogel exhibits excellent biocompatibility and good in vivo degradability, representing a reliable anti-adhesion barrier. It was found that the anti-C5aRab treatment exhibited outstanding performance in that it could inhibit peritoneal adhesions by reducing neutrophil infiltration and phosphorylated Smad2 expression. Furthermore, the method proposed by the authors can be considered an effective method for preventing peritoneal adhesions in clinical applications.

A summary of the last 5 years regarding the main characteristics and the biological properties of cellulose- and hemicellulose-based hydrogels that have applications in the field of tissue engineering is presented in Table 7.

## 6. Conclusions

Hydrogels based on cellulose and hemicellulose offer unique and exceptional advantages (biocompatibility and biodegradability) due to their intrinsic nature, but each material faces significant individual challenges, especially in terms of standardization of production methods, as well as of their mechanical and dimensional stability. Understanding the reaction mechanisms in the physical and chemical crosslinking processes that underlie the formation of these hydrogels is essential in order to obtain the expected strengths and specific properties. Moreover, in their design, a general approach is necessary that takes into account several factors such as the crosslinking density, homogeneity of structures, their morphology, etc. Research has evolved over time, and the key properties of hydrogels (mechanical and swelling properties) have seen significant progress.

Due to their fascinating and tunable properties, hydrogels have attracted major interest in biomedical applications, being considered perfect candidates for drug delivery, tissue engineering, wound dressing, etc. However, the global biomaterial market is constantly growing, which implies the emergence of more requirements and the need to find innovative solutions regarding the design of hydrogels with specific properties.

A new approach to hydrogel preparation is to design dynamic systems of non-covalent bonds/covalent bonds that allow the adaptation of the hydrogels’ properties (self-repair performance, shape memory and controlled stiffness). Self-healing and shape memory are two of the special properties of hydrogels that open up new possibilities for their use in biomedical applications, such as the concept of “close then heal”. This concept refers to the shape memory capacity of polymers, which promotes autonomous healing by uniting damaged sections. The use of self-healing approaches assisted by shape memory has recently found applications not only in TE and drug delivery but also in the preparation of bioinks and 4D-printed scaffolds.

Another direction concerns the exploration of new efficient crosslinking methods, such as one-step radiation-induced ecological methods, highly specific click chemistry, enzyme-induced methods, one-step multicomponent reactions (MCRs), i.e., the three-component Passerini reaction (P-3CR), etc. These methods allow for the attainment of tunable properties that can be used in applications requiring sustained release of therapeutic agents or in the fabrication of 3D-printed hydrogels for future exploitation from wound dressing to scaffolds for TE.

The incorporation of nanoparticles or bioactive proteins into hydrogels is an effective strategy not only to improve the stability of therapeutic agents but also to design hydrogels dedicated to specific applications such as topical administration to skin and soft tissues, wound healing and TE for bones and cartilage. An example, in this case, is that of cellulose-based hydrogels, which do not show antibacterial activity, and to improve this aspect, it is necessary to incorporate either antimicrobial agents (antibiotics, biological extracts or antimicrobial peptides) or antimicrobial nanoparticles (metal or metal oxide). In comparison to cellulose, hemicellulose shows properties such as immunological defense, inhibition of cell mutation and antioxidant properties (the specific case of xylan). However, in order to use hemicellulose-based hydrogels as antimicrobial materials, preclinical and clinical studies are still necessary. In addition, the functionalization of both cellulose and hemicellulose should be considered in order to increase the efficiency of bacterial control.

The integration of multi-stimuli-sensitive functions into cellulose- and hemicellulose-based hydrogels should be widely concerned, as they offer tunable and controllable properties upon environmental changes (physicochemical or biological stimuli). Applications of multi-stimuli-sensitive hydrogels (“smart” hydrogels), are oriented towards advanced wound healing, targeted drug delivery and complex TE, offering anti-inflammatory, antibacterial and regenerative effects. In the case of cellulose-based hydrogels, although significant progress has been made, there are still many challenges. Some of these are related to an unsatisfactory response time and relatively low sensitivity, which may affect pharmaceutical efficacy and diagnostic accuracy (for the case of drug delivery and disease diagnosis). Due to these inconsistencies, most of the research has remained at the laboratory scale. An exception exists for heat-sensitive cellulose-based hydrogels, which have entered industrial production. Regarding hemicellulose-based hydrogels, although there are promising prospects, their poor mechanical properties and low stability restrict their widespread application. A collapse of the hydrogel structure during use leads to poor drug encapsulation/release, while deformation or fracture under dynamic loading can affect the integrity and functionality of wound dressings or TE scaffolds.

Recent research is focused on 3D bioprinting techniques, which have revolutionized the field of drug delivery, allowing precise control of the structure and function of hydrogels. Cellulose-based hydrogels have found applications in 3D printing given their similarities in composition and microstructure to living tissues. However, the use of cellulose in bioprinting is still limited due to its poor solubility in common solvents. The use of hemicellulose-based hydrogels in 3D printing has attracted particular attention in recent years, and they are generally used by mixing hemicellulose with other polymers or by chemically modifying hemicelluloses.

The newest research direction is oriented towards obtaining “living materials” by integrating genetically modified cells inside biofabricated scaffolds, which would allow for dynamic, on-demand therapeutic release, opening new horizons towards personalized medicine. The use of genetically modified cellulose (*Komagataeibacter xylinus*) enhances therapeutic efficacy while minimizing systemic toxicity. This approach makes it particularly effective in treating cancer or chronic diseases, thus providing effective and personalized therapeutic solutions.

Although significant progress has been made in the research of cellulose- and hemicellulose-based hydrogels, there are still many challenges that await answers. The development of standardized protocols and environmentally friendly and cost-effective preparation techniques must be considered, as these are crucial aspects for large-scale production. In addition, compliance with GMP manufacturing processes, meeting the requirements of the US Food and Drug Administration (FDA) and the European Medicines Agency (EMA) and optimizing the marketing strategy are necessary to accelerate clinical applications and commercialization. Continuous advances in materials science will enable the development of hydrogels with improved performance, functionality and compatibility and will play a key role in achieving a healthier future.

## Figures and Tables

**Table 1:** A comparative analysis of the structure and properties of cellulose and hemicellulose.

Structure/Properties	Cellulose	Hemicellulose
Source Abundance	Highly available	Highly available
	Processing can be expensive	Less used
Structure	Linear	Branched
	β-(1→4)-linked glucose chains	Heterogeneous
Functional Groups	Hydroxyl	Hydroxyl
	–	Acetyl
Crystalline Structure	Crystalline	Amorphous
Chemical Reactivity	Low	Higher
	Stable polymer backbone	Contains various functional groups
Solubility	Insoluble in water (native)	Soluble in water/alkaline
	Its derivatives are more soluble	–
Swelling Capacity	High	Moderate to high
	Abundant hydroxyl groups	Varies with composition
Mechanical Strength	Strong	Moderate
Biodegradability	High	High

**Table 2:** The physical crosslinking methods, reaction mechanisms and properties of cellulose- and hemicellulose-based hydrogels.

Type of Hydrogel	Preparation Method	Hydrogel Composition and Structure		Hydrogel Characteristics	Ref.
		Composition	Network		
Cellulose hydrogels	Hydrogen bonding	- CMC/PA	- hydrogen bonds between CMC and PA.	- highly porous network; - fine and interconnected pores; - SDE = 374%.	[66]
		- Ox-HPC/CMCS	- H-bonds and Schiff base reaction.	- smaller and uniform pore sizes; - gelation time: 30 min (24% Ox-HPC/6% CMCS).	[67]
		- CNC/PAM/LMC	- dually crosslinked hydrophobic-associated hydrogels: - (i) hydrophobic-associated micelles based on LMC; - (ii) radical polymerization of Amm and LMC; - (iii) H-bonds: CNCs and hydrophobically associated hydrogel.	- fracture strain (1.5% CNCs): 34%; - fracture stress (1.5% CNCs): 342%; - sensitivity: GF = 19.25 at 700% strain; - dissipated energy: 10.9 kJ/m^3^; - conductivity: 22.97 mS/cm.	[68]
	Freeze–thaw method	- CNF/CNT	- composite aerogel; - freeze–thaw cycles: entanglement of CNTs and CNFs.	- ⍴ = 0.0519 g·cm^−3^; - average pore sizes: 19.32 nm; - surface area: 157.24 m^2^/g; - conductivity: 30.95 S/cm.	[71]
		- CNF/CNT	- anisotropic aerogel; - multiple unidirectional freeze–thaw cycles.	- ⍴ = 0.0296 g·cm^−3^; - porosity: 98.1–98.6%; - shrinkages: 11.03%; - stress: 329.8 kPa at 75% strain.	[72]
	Ionic interactions	- cotton cellulose/PVA	- ionic conductive hydrogels: - (i) hydrogen bonds: cellulose and PVA; - (ii) ionic interactions: Zn^2+^/Ca^2+^ and OH groups of cellulose.	- tensile strength: 0.30 MPa, - compressive strength: 2.05 MPa at 70% strain; - elongation at break: 130%; - conductivity of 8.16 S m^−1^.	[73]
		- CMC	- dual-crosslinked network: - (i) coordinated aqua-complexes by Al^3+^; - (ii) ionic crosslinking: aqua–complexes with the –COO^–^ groups of CMC and CA; - (iii) H-bonds: aqua–complexes with -COOH groups of CMC and CA.	- high thermal stability; - CMC–CA–Al hydrogels: absorption/exchange properties and pH-responsive behavior;- adsorption capacity Li^+^ ions = 50 mg/g sorbent; - adsorption capacity Cs^+^ ions = 3–12.2 mg/g sorbent.	[74]
		- PEDOT/SCNF/Al(TFSI)_3_/PAA/TOCNFs/Al^3+^ ions	- interpenetrating core−sheath-structured conductive nanofibers incorporated in a physically crosslinked polyelectrolyte; - hydrogel network: electrostatic interactions, H-bonds, hydrophobic interaction and static π-π stacking.	- conductivity: 7.1 S/m; - stretchability: 770%; - self-healing efficiency: 80% after 2 h at room temperature; - sensitivity: 0.14 kPa^−1^; - self-adhesion: 28 kPa (pigskin).	[75]
Hemicellulose hydrogels	Hydrogen bonding	- TA@HC/PAA	- PAA/TA@HC/Fe^3+^ hydrogel: - (i) H-bonds between hydrophilic groups of components; - (ii) reversible interfacial interactions with Fe^3+^ (metal complexes); - (iii) free radical polymerization of AA.	- tensile stress: 115 kPa; - deformation: 5600%; - toughness: 4400 kJ/m^3^; - adhesion strength: 8.5 kPa (pigskin), 12.6 kPa (plastic) and 17.8 kPa (paper).	[76]
		- HC/GO	- HC-based hydrogels: - (i) H-bonds: between the OH groups (HC) and carboxyl groups (GO); - (ii) hydrogen bonds: between the OH groups (HC) and OH groups (GO).	- interconnected 3D porous network; - excellent mechanical properties; - non-toxic and biocompatible for cells.	[77]
	Freeze–thaw method	- H/PPY/B/GL/PVA	- PVA/B–GL–H/PPY hydrogel: strong H-bonds between PVA, H/PPY and GL; - freeze–thaw method (2 cycles): a multifunctional conductive composite hydrogel.	- tensile strain: 1094.9%; - stress: 480.6 kPa; - compressive stress: 1635.4 kPa; - toughness: 2.82 MJ/m^3^.	[78]
	Ionic interactions	- CM–Hemi/N–CDs	- CM–Hemi@Ca hydrogel: ionic bonds between Ca^2+^ and the COO^–^ groups in CM-Hemi; - network of CM–Hemi@Ca–N–CD hydrogel: amide bonds between the COOH groups of CM–Hemi and the NH_2_ groups on the N–CD surface.	- pore size: 1.63–2.46 µm; - good binding interactions with proteins; - bond length: 1.92 A° (*Staphylococcus aureus* and *Candida albicans*); - bond length: 2.01 A° (*Escherichia coli*).	[79]

*Abbreviations:* AA—acrylic acid; Amm—acrylamide; B—borax; CA—citric acid; CMC—carboxymethyl cellulose; CMCS—carboxymethyl chitosan; CM–Hemi—carboxymethyl hemicellulose; CNCs—cellulose nanocrystals; CNF—cellulose nanofibril; CNT—carbon nanotube; GF—gauge factor; GL—glycerol; GO—graphene oxide; H—hemicellulose; HC—hemicellulose; LMC—lauryl methacrylate; N–CDs—nitrogen-doped carbon dots; Ox-HPC—oxidized hydroxypropyl cellulose; PA—phytic acid; PAA—poly(acrylic acid); PAM—polyacrylamide; PEDOT—poly(3,4-ethylenedioxythiophene); PPY—polypyrrole; PVA—polyvinyl alcohol; SCNFs—sulfonated cellulose nanofibers; TA@HC—tannic acid-modified hemicellulose nanoparticles; TOCNFs—TEMPO- oxidized CNFs.

**Table 3:** Summary of chemical crosslinking methods, reaction mechanisms and properties of cellulose- and hemicellulose-based hydrogels.

Type of Hydrogel	Preparation Method	Hydrogel Composition and Structure		Hydrogel Characteristics	Ref.
		Composition	Network		
Cellulose hydrogels	Ring-opening reactions	- cellulose	- cellulose-based hydrogels chemically crosslinking with different epoxy crosslinkers: (i) ECH, (ii) BDDE and (iii) TMPTGE.	- Q = 1030% (CE); 710%; 580% (CB); 470% (CT); - thermal stability: 372 °C (CE); 403 °C (CB); 388 °C (CT).	[103]
		- CNFs/PGO	- CNF-PGO complex: H-bonds; - PGC bio-nanosheets: physical self-assembly (freeze–thaw treatment of CNF-PGO); - PGCNSH hydrogel: chemical crosslinking of PGC bio-nanosheets with ECH.	- excellent mechanical performances; - long-term underwater mechanical stability; - electrical conductivity (30 days): >6 S/m; - sensitivity (GF): 1.76 in the strain range of 15–20%.	[104]
		- CI, CII or CIII	- cellulose-based hydrogels chemically crosslinked with ECH.	- Q = 2128% (H-CI); 2440% (H-CII); 1926% (H-CIII); - H-CIII: stronger and stiffer structure; - H-CII: softer, stable structure.	[105]
		- cellulose/CMC	- cellulose-based hydrogel chemically crosslinked with ECH.	- Q = 2055%; - maximum soil moisture: 36.5% (topsoil); 30.1% (wet clayey soil); 23.4% (sandy soil).	[106]
	Polymerization reactions	- CNF/PAAm/TA	- ionic organohydrogel PAAm-CNF-TA-NaCl: - (i) PAAm/CNF as hybrid skeleton; - (ii) TA-functionalized interface; - (iii) electrolytes (NaCl) dissolved in a binary solvent glycerol–water system.	- Young’s modulus: 23 kPa; - mechanical strength: 86 kPa; - ionic conductivity: 2.7 S/m; - self-adhesiveness: 103 N/m; - UV-blocking efficiency: 99.9%.	[107]
		- cellulose/PDMAEMA	- hydrogel: free radical polymerization of DMAEMA and MBA in cellulose solution.	- ERD (% of the initial Q): 8–10% (1-1); 24.5–32.5% (1-3); 40.0–44.8% (1-5).	[108]
		- cellulose–IBBr/NIPAM	- PNIPAM-*g*-Cell copolymer: photoinduced metal-free atom transfer radical polymerization; - cellulose–IBBr as macroinitiator and PTH as catalyst.	- high microporosity and well-defined structure; - pore size: 100 μm.	[109]
	Schiff base reaction	- OHEC/HEA/DSPO	- P(HEA-co-AAm): free radical polymerization of OHEC, AAM, HEA and DSPO; - P(HEA-co-AAm)-OHEC-DSPO composite hydrogel: - (i) Schiff base bonds: OHEC and P(HEA-co-AAm); - (ii) H-bonds: P(HEA-co-AAm) and OHEC; - (iii) boron–oxygen bridges: borax and HEA.	- dual stimuli-response hydrogels; - T: >93% (DSPO < 2 × 10^−4^ mM); - compressive strength: 8.5 MPa; - Q: increase with increasing pH values; - non-toxicity (L929 cells); - intelligent contact lenses.	[110]
		- DCMC/AG/PAA	- dual network hydrogels: - (i) first layer: Schiff reaction between DCMC and AG; - (ii) second layer: free radical polymerization of acrylic acid (AA).	- compressive strain: 50%; - tensile strain: 550%; - cyclic compression: ≥10,000 times; - GF = 8.1; - adhesion: ≥10 times.	[111]
		- OMCC/CMCS	- hydrogel synthesis based on Schiff base reaction.	- SR = 31.18 g/g (water, OMCM-22); - SR = 40 g/g (pH 9.0, OMCM-22); - stress: 25 kPa at 68% strain (OMCM-79).	[112]
Hemicellulose hydrogels	Ring-opening reactions	- CHC/CS	- hydrogel’s network: - (i) dissolving CHC and CS in an alkali/urea/water system; - (ii) chemical crosslinking of CHC and CS with ECH.	- pore size: 200 μm; - compressive strength: 325 kPa.	[113]
	Small molecule chemical crosslinking	- beech xylan/DTPA	- hydrogel: one-step reaction by covalent crosslinking of xylan.	- SR: >62; - SR depending on pH; - photocytotoxicity against *S. aureus*, *P. aeruginosa*, *E. coli* and *B. cereus.*	[114]
		- CMX/G/OCNT	- CMX-g-G/OCNT hydrogel: - (i) covalent amide bonds: CMX (–COOH groups) and G (–NH_2_ groups); intermolecular H-bonding; - (ii) salting-out treatment Na_3_cit; - (iii) doping with OCNT.	CMX-g-G hydrogel: - tensile strain: 1.54 MPa; - tensile stress, 324%; - toughness: 2.31 MJ m^−3^; CMX-g-G/OCNT hydrogel: - tensile strength: 1.63 MPa.	[115]
	Polymerization reactions	- CMX–DA/PAM/bentonite	- hydrogel’s network: - (i) free radical polymerization (PAM); - (ii) grafting of PAM onto the CMX-DA; - (ii) physical interactions: bentonite with polymer chains.	- SR: 14.39 g/g; - compressive strength: 218.29 kPa; - tensile strength: 42.17 kPa; - elongation at break: 436%; - adhesion properties (SFE, mJ/m^2^): 62.78 (glass), 64.87 (plastic) and 67.39 (metal).	[116]
		- xylan-MAH/GO-VTEO	- GO–VTEO–xylan–MAH hydrogel: free radical polymerization.	- SR depending on pH; - pH = 5 (the optimum pH for the adsorption of Cu^2+^ ions) - maximum adsorption of Cu^2+^: 228 mg/g.	[117]
		- HC/CSN/PAA	- HC-CSN-Fe^3+^ hydrogel: - (i) free radical copolymerization (PAA); - (ii) grafting of HC onto PAA; - (iii) complexing Fe^3+^ with the amino groups of CSN.	- water retention in the soil (48 h): 97.54%; - sustained urea release; - antioxidant performance: 76.75%; - UV blocking efficiency: 92.2%.	[118]
		- HC/Pam	- HC–g–Am–BIS–BT hydrogels prepared by free radical graft copolymerization (HC acts as a crosslinking agent): - (i) covalent bonds between HC and Am; - (ii) H-bonds between HC and Am–BIS–BT.	- uniform and regular network structure; - maximum MB adsorption: 140.66 mg/g;	[119]
		- DAGMA/PAM/HC	- PAM-DM-HC hydrogels: - (i) synthesis of DAGMA: from DA and GMA; - (ii) synthesis of the PAM: polymerization process of AM; - (iii) hydrogel: copolymerization process between PAM and HC.	- tensile strength: 0.34 MPa; - tensile strain: 2098.4%; - toughness: 3.79 ± 0.95 MJ/m^3^; Hydrogels with MXene: - strength: 0.51 MPa; - toughness: 5.95 ± 1.19 MJ/m^3^.	[120]
		- HC/NP/PAM/PAC	- P(AM-AC)-HC-NP hydrogel: - (i) non-covalent bonds and electrostatic interactions: between the amino groups (PAM), carboxyl groups (PAC) and catechol group (NP); - (ii) grafting copolymerization of HC with acrylamide (AM) and acrylic acid (AC).	- compressive strain: 88%; - compression stress: 650 kPa; - stable mechanical properties: after 1000 cycles of cyclic compression; - good adhesion on different surfaces; - stable electrochemical properties.	[121]
		- XH–LA/[P(AA-co-DMAEMA)]/sodium alginate	- composite hydrogel: a double network formed by alginate/Ca^2+^ and P(AA-co-DMAEMA) with hemicellulose-based nanoaggregates: - (i) vinyl-functionalized amphiphilic hemicellulose derivative (XH-LA-MA); - (ii) ionically crosslinked sodium alginate/Ca^2+^ network; - (iii) covalently crosslinked P(AA-co-DMEMA) network; - (iv) XH-LA-MA participates in the hydrogel through covalent bonds.	- denser pore structure; - robust mechanical stability; - sustained drug delivery in load-bearing environments.	[122]
		- XH/PAA/Fe_3_O_4_ MNPs	- Fe_3_O_4_@XH-Gel nanocomposite hydrogel: - (i) free radical polymerization of PAA; - (ii) grafting AA onto XH chains (PAA–Xylan–PAA); - (iii) crosslinking reaction between the polymers (Xylan–PEGDA–Xylan); - (iv) crosslinking reactions between PEGDA and PAA (Xylan–PEGDA–PAA).	- pH-responsive characteristics; - SR = 5.0 (pH = 2); - SR = 48.9 (pH = 8); - magnetic responsiveness characteristics.	[123]
	Schiff base reaction	- DAX/gelatin/PVOH	- PVOH/gelatin-DAX hydrogel (I–PGD): - (i) chemically crosslinked network: Schiff base reaction (DAX and gelatin); - (ii) physically crosslinked network: PVOH; - synthesis of the Hofmeister-enhanced conductive composite hydrogel (H–PGD): the induction of polymer chain aggregation by ions during salinization.	- tensile strength: 3.02 MPa; - elongation: 330.95%; - factor of roughly (60 times): 1.79 MPa; - ionic conductivity: 5.4 × 10^−3^ S/m; - electrode specific capacitance: 186 F/g.	[124]
	Thiol-ene reaction	- GGMMA/CNCs–SH	- GGMMA/CNCs-SH hydrogel: thiol-ene crosslinking reaction between GGMMA and CNCs–SH.	- mechanical stiffness: 12.35 kPa; - sustained release of Si and Ca ions/species.	[125]

*Abbreviations*: AA—acrylic acid; Aam—acrylamide; AG—amino gelatin; Am—acrylic monomers; BDDE—1,4-butanediol diglycidyl ether; BIBB—2-Bromoisobuturyl bromide; cellulose–IBBr—BIBB-modified cellulose; CHC—corn cob hemicellulose; CMC—carboxymethylcellulose; CMCS—carboxymethyl chitosan; CMX—carboxymethyl xylan; CMX–DA—dopamine-grafted carboxymethyl xylan; CNF—cellulose nanofibril; CS—chitosan; CSN—chitosan; CNCs-SH—thiolated cellulose nanocrystals; DA—dehydroabietic acid; DAGMA—DA derivative 2-methacryloyloxyisopropanol ester; DAX—dialdehyde xylan; DCMC—dialdehyde carboxymethyl cellulose; DMAEMA—N,N-dimethylaminoethyl methacrylate; DSPO—double bond-functionalized spirooxazine; DTPA—diethylenetriaminepentaacetic dianhydride; ECH—epichlorohydrin; ERD—equilibrium reswelling degree; Fe_3_O_4_ MNPs—Fe_3_O_4_ magnetic nanoparticles; G—gelatin; GF—gauge factor; GGMMA—methacrylate-modified O-acetyl-galactoglucomannan; GMA—glycidyl methacrylate; GO-VTEO—graphene oxide modified with vinyltriethoxysilane; HC—hemicellulose; HC-CSN-Fe^3+^—hemicellulose–chitosan–iron ion hydrogel; HEA—2-Hydroxyethyl acrylate; MB—methylene blue; MBA—N,N-methylenebis(acrylamide); Na3cit—sodium citrate dihydrate; NP—nanopolydopamine; OCNTs—hydroxyl carbon nanotubes; OHEC—oxidized hydroxyethylcellulose; OMCC—oxidized microcrystalline cellulose; P(HEA-co-AAm)—hydroxyethyl acrylate-co-acrylic amide; PAA—polyacrylic acid; PAAm—polyacrylamide; PAC—polyacrylic acid; PAM—polyacrylamide; PDMAEMA—poly(dimethylaminoethyl methacrylate); PGCNSH—PGC bio-nanosheet-assembled hydrogel; PGO—graphene oxide; PTH—10-Phenylphenothiazine; PVOH—polyvinyl alcohol; Q—swelling degree; SFE—surface free energy; SR—swelling rate; T—transparency; TA—tannic acid; TMPTGE—trimethylolpropane triglycidyl ether; XH—xylan hemicellulose; XH–LA—nanoaggregates of hemicellulose laurate; XH–LA–MA—nanoaggregates of xylan-rich hemicellulose laurate polymers; xylan-MAH—xylan modified with maleic anhydride.

**Table 4:** Advantages and disadvantages of cellulose- and hemicellulose-based hydrogels.

	Cellulose Hydrogels	Hemicellulose Hydrogels
Advantages	Most abundant natural polymer	Wide abundance in nature
	Biodegradability	Biodegradability
	Biocompatibility	Biocompatibility
	Non-toxicity	Non-toxicity
	Non-immunogenic	Non-immunogenic
	–	Anti-inflammatory properties
	–	Antioxidant properties
	–	Protective barrier against bacterial infection
	High swelling ability	Good swelling capacity
	Excellent mechanical properties	Low-to-moderate mechanical strength
	Cost-effectiveness	Cost-effectiveness
Disadvantages	Not biodegradable in human body (lack of specific enzyme)	Not biodegradable in human body (lack of specific enzyme)
	–	Lack of reproducibility (batch variability)
	–	Poor stability over time
	Low cell adhesion	Low cell adhesion
	Absence of antimicrobial properties	–

**Table 5:** Cellulose- and hemicellulose-based hydrogels in drug delivery applications.

Hydrogel Type	Hydrogel Network	Characteristics	Biological Properties		Ref.
			Drug Release	Biocompatibility/ Antibacterial/ Anti-Inflammatory	
Cellulose hydrogels	- Ox-HPC/CMCS	- physically crosslinked hydrogels; - injectable; - pH-responsive; - self-healing properties.	- drug: phenylalanine; - drug release: 60% (12.2 h, pH 7.4); - drug release: 60% (8.3 h, pH 6.8).	-	[67]
	- MC/PLA/EURFS100	- physically crosslinked hydrogels; - pH-responsive; - colon-targeting oral drug delivery.	- drug: GlcNAc; EURFS100/PLA (70/30 *w/w*)-MC (3% *w/v*); - drug release: 90% (29 h); - drug release: <20% (stomach and small intestine).	- L929 cells; - no cytotoxicity (hydrogels with or without drug, 1 day).	[167]
	- C/D	- chemically crosslinked hydrogels; - anti-inflammatory properties.	- BAC: PF; - BAC release: 45.01% (14 days, D hydrogel); - BAC release: 20.9% (14 days, C hydrogel).	- hVIC and hVEC; - cell viability: >80%; - anti-inflammatory effects: reduced interleukin IL-6 and chemokine MCP-1.	[165]
	- cellulose/PDMAEMA	- chemically crosslinked hydrogels.	- antibacterial properties: due to PDMAEMA and Ag^+^ ions; - Ag content: 4 wt% (1-3 CP); - Ag content: 3.1 wt% (1-5 CP).	-	[108]
	- Na.CMC	- physically crosslinked hydrogels; - transdermal drug delivery.	- drug: RTG; - drug release: 95% (120 min, pH 7.4, NS-HG). - drug release: >90% (15 min, pH 7.4, ME-HG).	- SD rats (male, 220 ± 20 g, 6-week-old); - ME-HG: induced skin erythema (2 days); - NS-HG: non-toxic, good biocompatibility.	[169]
	- cellulose–IBBr/NIPAM	- chemically crosslinked hydrogels; - injectable; - thermo-responsive.	- drug: DOX; - drug release (PNIPAM-*g*-Cell): stable for 240 h.	- L929 cells; - cell viability: >96.9% (100 mg/mL).	[109]
	- CMC/PVP	- chemically crosslinked hydrogels; - injectable; - self-healing properties.	- drug: 4-ASA; - drug release: 50% (pH 2); - drug release: 70% (pH 7.4).	- NIH-3T3 cells; - cell viability: 95% (5 days).	[166]
Hemicellulose hydrogels	- HC/GO	- physically crosslinked hydrogels; - pH-sensitive; - oral drug delivery.	- drug: VB12; - drug release: 30% (12 h, pH 1.7); - drug release: 85% (12 h, pH 7.4).	- HEK 293 T cells; - cell viability: >90% (0.05 mg/mL); - cell viability: 80% (0.1 mg/mL).	[77]
	- KGM/OHA	- physically crosslinked hydrogels.	- drug: EGCG; - drug release: 62.44 ± 1.97% (10 h, KO-3).	-	[175]
	- bagasse HC/PDA	- chemically crosslinked hydrogels; - pH-sensitive hydrogels.	- drug: methylene blue; - drug release: 62.82% (composite hydrogel); - drug release: 47.77% (hemicellulose hydrogel).	-	[170]
	- beech XH	- chemically crosslinked hydrogels; - good thermal stability (xyl-2).	- drug: TMPyP; - drug release: 88% (10 h, pH 7.4).	- photocytotoxicity against *S. aureus*, *P. aeruginosa*, *E. coli* and *B. cereus*; - dark conditions: no cytotoxicity; - light irradiation: inhibition *S. aureus* (20.7 ± 1.5 mm).	[114]
	- XH/PAA/Fe_3_O_4_ MNPs	- chemically crosslinked hydrogels; - pH/magnetic dual-responsive.	- drugs: acetylsalicylic acid and theophylline; - acetylsalicylic acid release: 90% (6 h, pH 7.4); - theophylline release: 80% (6 h, pH 7.4).	- L929 cells; - cell viability: 82.50% (5 wt% Fe_3_O_4_ MNPs); - cell viability: 111.73% (15 wt% Fe_3_O_4_ MNPs).	[123]
	- XH–LA/[P(AA-co-DMAEMA)]/sodium alginate	- chemically crosslinked hydrogels; - stimulus responsiveness.	- drug: Cur; - drug release: 93% (22 h, Cur/XH-LA); - drug release: 69–77% (48 h, Cur/XH-LA-MA).	- NIN/3T3 cells; - cell viability: 88% (CGel); - cell viability: 85% (XH-LA CGel, XH-LA-MA CGel).	[122]
	- CNCs–SH/GGMMA	- chemically crosslinked hydrogels; - injectable; - in situ sustained release of therapeutic ions.	- therapeutic agent: BaGNP and Cu-BaGNP; - therapeutic ions: Si, Ca, Cu; - Si ion release: rapid (3 days) and slow (the next 11 days); - Ca ion release: 14 days; - Cu ion release: no detectable.	-	[125]

*Abbreviations*: A BAC—bioactive compound; BIBB—2-Bromoisobuturyl bromide; C—cellulose; cellulose–IBBr—BIBB-modified cellulose; CMC—carboxymethyl cellulose; CMCS—carboxymethyl chitosan; CNCs-SH—thiolated cellulose nanocrystals; Cur—curcumin; D—dextran; DOX—doxorubicin; EGCG—epigallocatechin gallate; EURFS100—methyl acrylate–methyl methacrylate–methacrylic acid terpolymer (Eudragit^®^ FS100); GGMMA—methacrylate-modified O-acetyl-galactoglucomannan; GlcNAc—N-acetylglucosamine; GO—graphene oxide; HC—hemicellulose; hVECs—human valvular endothelial cells; hVICs—human valvular interstitial cells; KGM—konjac glucomannan; MC—methyl cellulose; ME-HG—microemulsion incorporated into hydrogel; Na.CMC—sodium carboxylmethyl cellulose; NIPAM—N-isopropylacrylamide; NS-HG—nanocrystalline suspensions incorporated into hydrogels; OHA—oxidized hyaluronic acid; Ox-HPC—oxidized hydroxypropyl cellulose; PAA—polyacrylic acid; PDA—polydopamine; PDMAEMA—poly(dimethylaminoethyl methacrylate); PFs—polyphenols; PLA—polylactic acid; PVP—poly(N-vinylpyrrolidone); RTG—rotigotine; SD—Sprague Dawley; TMPyP—5,10,15,20-tetrakis(1-methylpyridinium-4-yl)porphyrin tetraiodide; VB12—Vitamin B 12; XH—xylan hemicellulose; XH–LA—nanoaggregates of hemicellulose laurate; XH-LA-MA—xylan-rich hemicellulose laurate methacrylate.

**Table 6:** Cellulose- and hemicellulose-based hydrogels in wound healing applications.

Hydrogel Type	Hydrogel Network	Characteristics	Biological Properties		Ref.
			Biocompatibility/ Wound Healing	Drug Release/Antimicrobial/ Anti-Inflammatory	
Cellulose hydrogels	- cellulose	- physically crosslinked hydrogels; - antimicrobial activity.	- ex vivo model: pig skin; - inhibit bacteria growth: pigskin covered with F53 (72 h).	- antibacterial activities: against *E. coli* and *S. aureus*; - inhibit bacteria growth: F53 (pitahaya) and F12 (tomato).	[186]
	- C/LE	- chemically crosslinked hydrogels; - antimicrobial activity; - adhesion properties; - oral dressings.	- hGF cells; - cell viability: >100% (CLE samples); - cell viability: >99% (P-CLE samples).	- drug: PrHy; - drug release: 68% PrHy (1440 min, P-CLE 7); - drug release: 34% PrHy (1440 min, P-CLE); - antimicrobial activity: against *E. coli* and *S. aureus*.	[143]
	- OMCC/CMCS	- chemically crosslinked hydrogels; - pH sensitivity; - self-healing properties; - hemostatic effects.	- GES-1 cell; - cell viability: >85% (non-toxicity); - in vitro blood compatibility: human blood; - BCI: 0.037 (30 min).	- BAC: rutin; - the entrapment efficiency: 45.76% (OMCM-54); - BAC release: 47.77% (1 h, pH 7.0, OMCM-54); - BAC release: 90.22% (12 h).	[112]
	- BC/ PVA	- chemically crosslinked hydrogels; - antimicrobial activity.	- L929 cells; - cell viability: 96.9% (PBC4 and PBC5); - cell viability: 86% (PBC3).	- antibacterial activities: against *E. coli* and *S. aureus*; - inhibition zone diam.: 3 cm (*S. aureus*) and 2.6 cm (*E. coli*).	[183]
	- PDA@BC/ PVA via PB.	- chemically crosslinked hydrogels; - antibacterial activity; - tissue adhesiveness; - self-healing property.	- L929 cells; - cell viability: >85% (24 h); - in vivo model: Kunming mice (female, 5–6 weeks); - wound closure: 97.1% (PB-PDA@BC/Doxy, 14 days).	- drug: Doxy; - drug release: 88.3% (42 h, PB-PDA@BC1.5); - adhesion strength to skins: 4.7 ± 0.3 to 5.4 ± 0.4 kPa; - antimicrobial activity: 94% (against *E. coli* and *S. aureus)*.	[187]
	- DBC/ QCS	- chemically crosslinked hydrogels; - antibacterial activity.	- L929 cells; - cell viability: >90%; - in vivo model: SD rats (female, 6–8 weeks); - wound healing: 14 days (thicker granulation).	- drug: PFD; - drug release: slowly within 14 days (DBC/QCS/PFD); - antibacterial activity: against *E. coli* (80.8%) and *S. aureus* (81.3%).	[188]
Hemicellulose hydrogels	- xylan-PAA-TA	- chemically crosslinked hydrogels; - adhesion property; - antioxidant activity; - antibacterial ability.	- NIH-3T3 cells; - xylan-PAA-TA hydrogels showed the best cell affinity.	- antibacterial activity: against *S. aureus*; - antioxidant activity: 94.12% (xylan-PAA-TA3).	[189]
	- ARX/CS/rGO	- chemically crosslinked hydrogels; - pH-responsive; - antibacterial activity.	- MC3T3-E1 cells; - cell viability: 91% (ATC-4); - increased cell adhesion and proliferation.	- drug: silver-sulphadiazine; - drug release: 93.1% (pH 7.4) and 58.3% (pH 6.4); - antimicrobial activity: against *P. aeruginosa*, *E. faecalis* and *E. coli*.	[190]
	- XG/ BC/ D	- physically crosslinked hydrogels.	- male Sprague Dawley mice (7–8 weeks old); - wound healing area: 98.3% (14 days, BC-D); - wound healing area: 96.98% (14 days, BC-XG-D); - tissue thickness: 15.4 mm (BC-D) and 15.2 mm (BC-XG-D).	-	[191]
	- XG/QCS/PVA/COL	- chemically crosslinked hydrogels; - antibacterial activity; - promote angiogenesis.	- L929 and HaCaT cells; - cell viability: >100% (PCQX-M); - average proliferation rates: 192.99% (HaCaT) and 133.83% (L929); - in vivo model: BALB/c mice (male, 8 weeks old); - formation of granulation tissue deposition of collagen.	- antibacterial activity: against *E. coli* (96.74%, PCQX-M) and *S. aureus* (99.49%, PCQX-M).	[192]
	- XG/F127DA	- chemically crosslinked hydrogels; - antibacterial activity; -anti-inflammatory properties; - promote angiogenesis.	- L929 cells; - cell viability: 95%; - in vivo model: 45 male C57BL/6 mice; - reduce the production of pro-inflammatory cytokines (IFN-γ, TNF-α, IL-1β, IL-6 and IL-12); - wound closure rate: 98.30 ± 1.83% (3 days).	- antimicrobial activity: against *S. aureus* and *P. aeruginosa*.	[193]
	- XG/OP	- chemically crosslinked hydrogels; - injectability; - self-healing properties; - angiogenic activity.	- L929 and HUVECs cells; - cell viability: 110.6% (L929 cell) and >80% (HUVECs); - in vivo model: male SD rats (8–10 weeks age); - average healing rate: 94.5% (14 days).	- in vitro antioxidant capacity (the scavenging rate of DPPH): 73.9%; - anti-inflammatory properties; - hemolysis rate: 0.7% (hemostatic properties).	[194]
	- OXG/CSMA	- chemically crosslinked hydrogels; - injectability; - self-healing capability.	- NIH/3T3; - cell viability: 214.78 ± 6.49% (XC^UV^); - ICR mice (male, 8-week-old); - wound closure rate: 73.71% ± 3.54 (XC^UV^, 5 days); - epidermis thicknesses: 49.76 ± 6.71 μm (XC^UV^, 21 days); - granulation tissue: 247.84 ± 7.83 μm (XC^UV^, 5 days).	-	[195]
	- KGM/HA	- chemically crosslinked hydrogels; -antibacterial properties; - anti-biofilm efficacy.	- NIH/3T3 cells; - cell viability: 91.6 ± 4.55% (HAKGM, 72 h); - burn wound healing ratio: 98 ± 4.9% (HAKGM, 12 days); - burn wound shrinking rate: 90% (12 days).	- antimicrobial activity: against *E. coli* (75%) and *S. aureus* (98%); - inhibition of biofilm formation: 92.4 ± 4.3% (HAKGM).	[204]
	- KGM/GA	- chemically crosslinked hydrogels; - antioxidant properties.	- L929 cells, Raw264.7 cells; - cell viability: 95% (L929); - cell viability: 80% (Raw 264.7); - wound healing area: 31.1 ± 0.85% (KGM-GA, 3 days); - KGM-GA has the ability to regulate M2 macrophages.	- H_2_O_2_ scavenging activity: KGM-GA showed excellent ROS scavenging activities; - intracellular ROS level: after treatment with KGM-GA, the ROS in L929 cells was reduced from 35% to less than 20%.	[205]
	- GA/PVA	- chemically crosslinked hydrogels; - anti-inflammatory effects.	- fibroblasts, monocytes and osteoblasts; - cell viability: 185% (fibroblasts, 48 h, PVA/GA = 80:20); - cell viability: 133% (osteoblasts, 48 h, PVA/GA = 70:30); - PVA/GA = 80:20: decreasing MCP-1 cytokine secretion.	- antibacterial activity: against *E. coli* (52 to 72%).	[206]
	- GM/ κC; - lactoferrin or Cramoll.	- physically crosslinked hydrogels; - topical sustained release system.	- Wistar albino rats (Male, 8–10 weeks); - reduction in lesion area (14 days): 36.05% (HC), 45.49% (HL) and 66.10% (HLC). - HLC: a better level of epithelial recovery with typical collagen distribution, stratified re-epithelialization and thicker keratinization layer formation.	- biomolecule (Cramoll and lactoferrin) release: optimized lesion contraction.	[209]
	- OGM/HA/AM/MEDA	- chemically crosslinked hydrogels; - self-healing properties; - antibacterial performance.	- GES-1 cells; - cell viability: 100% (72 h); - SD rats (male, 6–8 weeks); - reduction in wound area: 90% (O_4_H_4_M_4_A, 7 days); - skin damage area: 0.14% (14 days).	- antibacterial activity (O_4_H_4_M_4_A): against *E. coli* and *S. aureus*; - OD_600_: 0.04 (*S. aureus*) and 0.05 (*E. coli*).	[210]

*Abbreviations*: AM—acrylamide; ARX—arabinoxylan; BAC—bioactive compound; Ba BC—bacterial cellulose; BCI—blood clotting index; C—cellulose; CMCS—carboxymethyl chitosan; COL—collagen; CS—chitosan; CSMA—amino residues of methacrylated chitosan; D—dextran; DBC—dialdehyde bacterial cellulose; Doxy—doxycycline; F127DA—pluronic F127 diacrylate; GA—gallic acid; GM—galactomannan; HA—hyaluronic acid; HaCaT—human immortalized keratinocytes; hGFs—human gingival fibroblasts; HUVECs—human umbilical vein endothelial cells; KGM—konjac glucomannan; L929—mouse fibroblast; LE—chemically modified lignin; MC3T3-E1—mouse pre-osteoblast cells; MEDA—[2-(methacryloyloxy)ethyl] dimethyl-(3-sulfopropyl) ammonium hydroxide; NIH/3T3—mouse fibroblast cells; OGM—aldehyde-grafted galactomannan; OMCC—microcrystalline cellulose; OP—okra polysaccharide; OXG—oxidized xyloglucan; PAA—poly (acrylic acid); PB—dynamic borate; PDA@BC—bacterial cellulose decorated with polydopamine; PDMAEMA—poly(dimethylaminoethyl methacrylate); PFD—drug pirfenidone; PrHy—procaine hydrochloride; PVA—polyvinyl alcohol; QCS—quaternized chitosan; rGO—reduced graphene oxide; SD—Sprague Dawley; TA—tannic acid; XG—xyloglucan; κC—κ-carrageenan.

**Table 7:** Cellulose- and hemicellulose-based hydrogels in tissue engineering applications.

Hydrogel Type	Hydrogel Network	Characteristics	Biological Properties	Ref.
			Biocompatibility/Anti-Inflammatory/ Drug Release/Antibacterial	
Cellulose hydrogels	- oxidized BC (OBCS)/AuNPs	- thermosensitive hydrogel; - injectable; - self-healing capacity; - heart TE applications.	- H9C2 cells; - cell viability: >92% (14 days); - self-healing efficiency: 80%; - electrical conductivity: 0.03 to 0.6 S/m; - BPM = 47; - FPA = 5.47 μv.	[222]
	- TEMPO—oxidized cellulose (TCNF)/ALG	- potential bioinks; - 3D-bioprinted meniscus-like tissue constructs; - fibrocartilage-like tissue.	- hMFC cells; - hMFC-deposited ECM was observed in bioinks (6 weeks); - TCNF/ALG bioinks (7030, 8020): support the development of a more inner meniscus fibrocartilage-like phenotype (presence of sGAG, collagen I and II); - COL (bovine) bioink: a more outer meniscus phenotype.	[219]
	- TEMPO-modified NCC (mNCC)/MeGel	- 3D-bioprinted self-standing tubular structure; - TE heart valves.	- HADMSC cells; - cell viability: >7 days; - the most cell spreading: 2.0 mNG (mNCC-MeGel); - HADMSC encapsulated within mNG: decreased αSMA expression and increased vimentin and aggrecan expression.	[226]
	- TEMPO- oxidized CNF (TOCN)/SCNT	- myocardial TE scaffold.	- L929 cells and H9C2 cells; - RGR of L929: 95% (>5 days)—good biocompatibility and non-cytotoxicity to myocardial cells; - growth of H9C2 cardiomyocyte (7 days): significantly increasing numbers of cells; - conductivity: 5.2 × 10^−6^ to 6.2 × 10^−2^ S/cm.	[227]
	- TEMPO—oxidized CNF (TOCN)/PPy	- myocardial TE scaffold.	- H9C2 cells and L929 fibroblasts; - RGR of L929: >90% (3 days)—composite conductive hydrogel has no cytotoxicity; - growth of H9C2 cardiomyocyte (7 days): the number of cells increased and interconnected to form a network; - hydrogel significantly increased the expression levels of cardiac-specific proteins cTnT and cx-43.	[228]
	- TEMPO-oxidized BC (m-TOBC)/MSN	- bioactive hydrogel scaffolds for bone defect repair; - bone tissue reconstruction and repair.	- HUVEC cells; - cell proliferation rate: the highest for GelMA/m-TOBC/DMSN; - wound closure rate: 65.5% (GelMA/m-TOBC/DMSN, 24 h); - enhanced in vitro mineralized matrix deposition and osteoblastic alkaline phosphatase expression; - promote angiogenesis (enhanced cell migration, tube formation and upregulated angiogenic gene expression levels).	[229]
	- HPMC/CS/Gyl	- osteogenesis capacity; - thermosensitive hydrogel; - antibacterial characteristics; - orthopedic implants.	- MC3T3-E1 osteoblasts and RAW264.7 cells; - released Gly: stimulated macrophage polarization to the pro-inflammatory M1 phenotype; - enhanced osteogenesis and immunoregulatory antibacterial activities; - drug: Sim; - Sim amount release (10 h): Sim@CGHH(sol) much higher than Sim@CGHH (gel); - antimicrobial activity: against *E. coli* (75%) and *S. aureus* (98%); - in vivo antibacterial activities: Sim@CGHH has significantly higher antibacterial activity.	[221]
	- HEC/CS/BGP/CNCs	- 3D complex bone tissue-engineered constructs; - bone TE applications.	- MC3T3-E1 cells; - good cell viability and proliferation in the bioprinted scaffolds (7 days); - CS + 1.5% CNC scaffolds exhibited the fastest osteogenesis; - CS−CNC scaffold: accelerated activity of alkaline phosphatase, calcium mineralization and collagen formation in ECM.	[230]
	- CMC/ALG/HEMA/AA/(PVA)	- cardiac TE applications.	- H9c2 cells; - cell viability: 101.48 ± 1.89% (CAHA2A) and 89.61 ± 3.22% (CAHA2AP); - ECM content (15 days): 1.84± 0.23% (CAHA2A) and 1.88 ± 0.08% (CAHA2AP); - both hydrogel scaffolds: deliver cells to the local environment and promote anterograde and retrograde movement.	[231]
	- regenerated cellulose (rCL)/CS	- electrospun regenerated nanofibers; - bone TE applications.	- MC3T3-E1 cells; - rCL/CS enhanced MC3T3-E1 cell attachment and proliferation; - rCL/CS improved bioactivity (Ca/P ratio = 1.83); - rCL/CS increased ALP and ARS activity.	[232]
	- CNCs/Hap/pectin	- bone regeneration applications.	- L929 cells; - all hydrogels are non-toxic and biocompatible; - biomimetic method: HAp formation similar to biological hydroxyapatite.	[233]
	- CNCs/TA/AgNPs (CNCs/TA@AgNPs)/PVA/PDA@HAP	- bioactive hydrogel; - antibacterial properties; - osteogenic capabilities; - bone TE applications.	- BMSC cells; - cell viability: 95%; - PVA-Ag-PHA: supports BMSC adhesion and proliferation, and enhances the osteogenic differentiation of cells; - Sprague Dawley rats (3 weeks old); - PVA-Ag-PHA: exhibited a large amount of newly forming bone in the center of the bone defects (after implanting for 4 weeks); - PVA-Ag-PHA: promotes bone tissue healing over 8 weeks; - antimicrobial activity: against *S. aureus* and *E. coli.*	[234]
	- CNF/PVA/GMA	- soft and flexible hydrogel; - injectable; - meniscus TE applications.	- CSPCs; - cell viability (24 h): >95% (PVA-g-GMA and PVA-g-MA/CNF injectable hydrogels) is noncytotoxic; - cell viability (14 days): 81% (10%PVA-g-GMA/0.5%CNF) and 78% (10%PVA-g-GMA/0.7%CNF)—good cell adhesion and viability in the long-term culture.	[235]
Hemicellulose hydrogels	- Xylan/CS/Hap/(GO/RGO)	- bone TE applications.	- MG-63 cells; - X/C/HAp/0.5%RGO: the highest cell viability; - samples with GO or RGO content: capable of osteointegration; - samples with RGO content: larger apatite morphology.	[224]
	- mXG/anti-C5aRab	- prevent postoperative peritoneal adhesions.	- HPMC cells; - cell viability: 100% (48 h); - KM mice (6 weeks old); - mXG/anti-C5aRab hydrogel: provided an adhesion barrier while preventing an excessive inflammatory response.	[225]

*Abbreviations*: AA—acrylic acid; AgNPs—silver nanoparticles; αSMA—alpha smooth muscle actin; ALG—alginate; anti-C5aRab—anti-C5a receptor antibody; A AuNPs—gold nanoparticles; BC—bacterial cellulose; BGP—β glycerophosphate; BPM—number of beats per minute; BMSCs—bone marrow mesenchymal stem cells; CMC—carboxymethyl cellulose; CNCs—cellulose nanocrystals; CNF—cellulose nanofiber; CS—chitosan; CSPCs—human cartilage stem/progenitor cells; FPA—field potential amplitude; GMA—glycidyl methacrylate; GO—graphene oxide; Gyl—glycerin; H9C2—rat cardiomyocyte cell line; HADMSCs—human adipose-derived mesenchymal stem cells; HAp—hydroxyapatite; HEC- hydroxyethyl cellulose; HEMA—hydroxyethyl methacrylic acid; hMFCs—human meniscus fibrochondrocytes; HPMC—hydroxypropyl methyl cellulose; HUVECs—human umbilical vein endothelial cells; L929—mouse fibroblast cell line; MeGel—methacrylated gelatin; mNCC—TEMPO-modified nanocrystalline cellulose; MSNs—nanofibers and mesoporous silica nanoparticles; m-TOBC—TEMPO-oxidized bacterial cellulose; mXG—xyloglucan derivative; OBCS—oxidized bacterial cellulose; PDA@HAP—polydopamine-modified hydroxyapatite; PPy—polypyrrole; PVA—poly(vinyl alcohol); rCL—regenerated cellulose; RGO—reduced graphene oxide; RGR—relative growth rate; SCNTs—sulfonated carbon nanotubes; Sim—simvastatin; TA—tannic acid; TCNF—TEMPO (2,2,6,6-tetramethylpiperidine-1-oxyl)-oxidized cellulose; TOCNs—TEMPO-oxidized cellulose nanofibrils.

## Data Availability

No new data were created or analyzed in this study.
